# Combining Persuasive System Design Principles and Behavior Change Techniques in Digital Interventions Supporting Long-term Weight Loss Maintenance: Design and Development of eCHANGE

**DOI:** 10.2196/37372

**Published:** 2022-05-27

**Authors:** Rikke Aune Asbjørnsen, Jøran Hjelmesæth, Mirjam Lien Smedsrød, Jobke Wentzel, Marianne Ollivier, Matthew M Clark, Julia E W C van Gemert-Pijnen, Lise Solberg Nes

**Affiliations:** 1 Center for eHealth and Wellbeing Research Department of Psychology, Health and Technology University of Twente Enschede Netherlands; 2 Research and Innovation Department Vestfold Hospital Trust Tønsberg Norway; 3 Department of Digital Health Research Division of Medicine Oslo University Hospital Oslo Norway; 4 Morbid Obesity Center Vestfold Hospital Trust Tønsberg Norway; 5 Department of Endocrinology, Morbid Obesity and Preventive Medicine Institute of Clinical Medicine University of Oslo Oslo Norway; 6 Collaborative Care Unit Sørlandet Hospital Trust Kristiansand Norway; 7 Research Group IT Innovations in Health Care Windesheim University of Applied Sciences Zwolle Netherlands; 8 Department of Psychiatry & Psychology College of Medicine & Science Mayo Clinic Rochester, MN United States; 9 University of Waterloo Waterloo, ON Canada; 10 Institute of Clinical Medicine Faculty of Medicine University of Oslo Oslo Norway

**Keywords:** eHealth, weight loss maintenance, behavior change, persuasive technology, digital health interventions, design thinking, co-design, Agile development, human-centered design, mobile phone

## Abstract

**Background:**

Long-term weight maintenance after weight loss is challenging, and innovative solutions are required. Digital technologies can support behavior change and, therefore, have the potential to be an effective tool for weight loss maintenance. However, to create meaningful and effective digital behavior change interventions that support end user values and needs, a combination of persuasive system design (PSD) principles and behavior change techniques (BCTs) might be needed.

**Objective:**

This study aimed to investigate how an evidence-informed digital behavior change intervention can be designed and developed by combining PSD principles and BCTs into design features to support end user values and needs for long-term weight loss maintenance.

**Methods:**

This study presents a concept for how PSD principles and BCTs can be translated into design features by combining design thinking and Agile methods to develop and deliver an evidence-informed digital behavior change intervention aimed at supporting weight maintenance. Overall, 45 stakeholders participated in the systematic and iterative development process comprising co-design workshops, prototyping, Agile development, and usability testing. This included prospective end users (n=17, 38%; ie, people with obesity who had lost ≥8% of their weight), health care providers (n=9, 20%), healthy volunteers (n=4, 9%), a service designer (n=1, 2%), and stakeholders from the multidisciplinary research and development team (n=14, 31%; ie, software developers; digital designers; and eHealth, behavior change, and obesity experts). Stakeholder input on how to operationalize the design features and optimize the technology was examined through formative evaluation and qualitative analyses using rapid and in-depth analysis approaches.

**Results:**

A total of 17 design features combining PSD principles and BCTs were identified as important to support end user values and needs based on stakeholder input during the design and development of eCHANGE, a digital intervention to support long-term weight loss maintenance. The design features were combined into 4 main intervention components: *Week Plan*, *My Overview*, *Knowledge and Skills*, and *Virtual Coach and Smart Feedback System*. To support a healthy lifestyle and continued behavior change to maintain weight, PSD principles such as *tailoring*, *personalization*, *self-monitoring*, *reminders*, *rewards*, *rehearsal*, *praise*, and *suggestions* were combined and implemented into the design features together with BCTs from the clusters of *goals and planning*, *feedback and monitoring*, *social support*, *repetition and substitution*, *shaping knowledge*, *natural consequences*, *associations, antecedents, identity*, and *self-belief.*

**Conclusions:**

Combining and implementing PSD principles and BCTs in digital interventions aimed at supporting sustainable behavior change may contribute to the design of engaging and motivating interventions in line with end user values and needs. As such, the design and development of the eCHANGE intervention can provide valuable input for future design and tailoring of evidence-informed digital interventions, even beyond digital interventions in support of health behavior change and long-term weight loss maintenance.

**Trial Registration:**

ClinicalTrials.gov NCT04537988; https://clinicaltrials.gov/ct2/show/NCT04537988

## Introduction

### Background

Healthy lifestyle and behavior changes are difficult to initiate but even more challenging to sustain over time [1]. There has been an increase in lifestyle-related diseases such as obesity, cardiovascular diseases, and diabetes worldwide [2-4]. The estimated number of people with obesity nearly tripled between 1975 and 2016, with >650 million adults with obesity (BMI≥30 kg/m²) worldwide in 2016 [5]. Similarly, the number of people with cardiovascular diseases increased by >50% in the period from 1990 to 2019 [6], and people living with diabetes increased by >60% in the period from 2009 to 2019 [7].

For people with obesity, weight loss may be difficult; however, maintaining weight after weight loss appears to be even more of a challenge [8], and several factors (eg, environmental, biological, behavioral, and cognitive) [9-12] contribute to the complexity of this health problem. The impact and burden of obesity on an individual are substantial and multifaceted, as it can affect both health and well-being, with an increased risk of medical conditions, premature mortality, and reduced quality of life [13,14].

### The Challenge of Weight Loss Maintenance

Lifestyle interventions focusing on diet and physical activity, as well as behavioral and cognitive strategies, are widely recommended for obesity management [12,15-20]. However, even when weight loss is achieved, mechanisms such as increased hunger, reduced energy expenditure, and reduced satiety frequently contribute weight regain [10,17]. As much as 30% to 50% of the initial weight that is lost during lifestyle interventions is often regained during the subsequent 2 to 3 years [21], and few manage to maintain their lost weight in the long term [22]. With several factors contributing to weight regain [9,10,23], solving the weight loss maintenance challenge appears to be a complicated endeavor.

Health behaviors and self-regulation play central roles in weight loss and weight loss maintenance [24,25]. Implementing sustainable and feasible behavior change and self-regulation strategies into daily life takes time and effort, and finding ways of initiating and maintaining behavior change over a long period is a complex undertaking [26].

Health behaviors also change over time and are often person and context related (eg, individual motives, habits, and social and environmental factors) [23,26]. With many people failing to maintain weight after initial weight loss, innovative approaches for long-term behavior change and weight loss maintenance are needed [10,27].

### Advantages and Challenges of Digital Interventions

Digital interventions are increasingly being used to promote healthy lifestyles and improve health outcomes [28-31] and can be an accessible and feasible way of supporting behavior change through its availability and scalability. A digital health (ie, eHealth) intervention can be defined as a digital technology focusing on intervening in an existing situation aiming to change health behavior [29]. The potential of evidence-informed digital technologies supporting weight loss maintenance may be significant, as digital interventions can overcome the *time and place barrier* and adapt to a person’s context, needs, and preferences [32], consequently offering people the support needed for sustained behavior change [29,33].

Research exploring technologies for weight loss maintenance support is still at an early stage, and little is known about their long-term effectiveness, as few evidence-based eHealth interventions are available [34,35]. In addition, little is known about potential changes in user needs over time and how digital technologies can support end users in maintaining weight in an optimal manner for long-term behavior change [32]. In fact, knowledge is lacking on how to translate persuasive system design (PSD) principles and behavior change techniques (BCTs) into design features when developing digital interventions aimed at facilitating continued health behavior change and weight loss maintenance [35].

### Development of Innovative Solutions for Sustainable Behavior Change

An important factor for the success of digital health research, development, and implementation is the early involvement of end users and other key stakeholders in the design and formative evaluation of a product or technology [29,36-39]. Human-centered design approaches such as design thinking [40,41] can be combined with principles from Agile software development [42], stimulating the *collective creativity* of end users and other key stakeholders (eg, designers, developers, researchers, and experts) for the rapid development and evaluation of digital health interventions [31,43-45].

Design thinking is an approach that emphasizes understanding and empathy with end users, multidisciplinary collaboration, and iterative involvement of stakeholders through generation of creative ideas and action-oriented rapid prototyping to create desirable, feasible, and viable innovative solutions [40,46]. Agile software development is a flexible approach that emphasizes active stakeholder involvement through rapid iterations to test assumptions and validate possible solutions to quickly learn and adapt to changes in needs [42]. Combining these design and development methods could therefore be a time- and cost-effective approach to explore and validate whether an innovative solution is desirable (ie, what users and stakeholders want) [40,42,43,47] and whether it solves the right problem (ie, meet user and stakeholder needs) for a *problem-solution fit [48].*

### Translating PSD Principles and BCTs Into Design Features

To support long-lasting behavior change through digital technologies, the design needs to support the user in adopting sustainable behaviors related to their individual goals and values [31,32]. Although goals relate to something a person would like to achieve, values can refer to *what a person considers important in life* [49], reflecting end users’ ideals or interests [50]. As such, values can be defined as the *main drivers of behaviors or high-level needs* [32]*.* Therefore, finding a *solution* that can help address end users’ goals by taking their key values and needs into account is important to create meaningful and effective eHealth technologies in support of continued health behavior change.

Complex interventions, such as digital behavior change interventions, usually consist of many active ingredients or interactive components [51-54] and can be designed to facilitate motivation and adherence to healthy behaviors [35]. PSD principles and BCTs can be such *active ingredients* or *building blocks* of digital behavior change interventions. To design motivating and effective digital technologies, integrated PSD principles and BCTs should match end user values and needs [32]. PSD principles are designed to influence users’ attitudes and behaviors [55] and can be applied to match user profiles to motivate and trigger health behavior change in the design of technologies [35,55,56]. By contrast, BCTs are designed to alter or redirect causal processes that regulate behavior [51,52], can be applied to any intervention focusing on behavior change to improve the health and well-being of people [29], and can be applied to daily life without technology [32]. As such, the PSD principles and BCTs overlap and complement each other [29,35]. Theoretical principles from the PSD model by Oinas-Kukkonen [55] and BCTs from the Behavior Change Taxonomy by Michie et al [52] can be translated into design features during the design and development of digital behavior change interventions to meet end user values and needs [29,32]. Such combined PSD [55] and BCT [52] features can be embedded in a digital application *with the specific aim of forming, altering, or reinforcing healthy attitudes and behaviors* [57].

However, research involving digital behavior change interventions often fails to clearly show how theories and techniques of behavior change have been combined and applied to design and practical delivery forms [31,35,57-61]. There appears to be a lack of theoretical frameworks and specifications of design features when reporting on digital interventions. It is often unclear which design features, PSD principles, and behavior change strategies are most effective in meeting end user values and needs and how they influence health-related outcomes, including weight loss maintenance [29,35,52,62-64].

In response to these gaps, the current research group performed a scoping review and a qualitative study aimed at identifying PSD principles and BCTs from eHealth interventions applied in existing weight loss maintenance research [35], as well as key values and needs related to *what* people want for maintaining weight and *why* [32]. The scoping review [35] identified PSD principles [55] (eg, *self-monitoring*, *reminders*, *rewards*, *tailoring*, *personalization*, and *praise*) and BCT clusters [52] (eg, *feedback and monitoring*, *goals and planning*, *repetition and substitutions*, *social support*, *associations*, and *shaping knowledge*) applied in eHealth interventions to stimulate adherence, motivation, and weight loss maintenance. The technology characteristics of existing eHealth weight loss maintenance interventions were usually supported by mobile phone technology, sometimes in combination with an activity tracker and wireless scale [35]. The subsequent qualitative study (ie, individual and focus group interviews) identified key end user values of people with the aim of maintaining weight loss in the long term (ie, *autonomy, self-management, motivation, personalized care, happiness, health, feel supported*, and *positive self-image*), as well as PSD principles and BCTs that might be essential to include in eHealth interventions to meet end user values and needs [32]. The findings indicated that the most successful and promising eHealth weight loss maintenance interventions entailed a combination of both PSD principles and BCTs [32,35]. The studies overlapped in findings [32,35]; however, some less frequently applied PSD principles (eg, *rehearsal*) and BCTs (eg, *identity, self-belief*, and *natural consequences*) were identified in the qualitative study, which might be of importance to support end user values and needs to prevent weight regain in the long term [32]. The qualitative study also highlighted the *tailoring* and *personalization* of digital interventions to address the often multifaceted and dynamic changes in individual needs over time (eg, related to behaviors, thoughts, and emotions) for continued health behavior change [32].

The results from these recent studies strongly suggest that the process of translating end user values into *design features*, as well as the exploration of how PSD principles and BCTs can be combined and implemented, are important parts of the design and development processes when aiming to create motivating and engaging digital interventions to support sustained behavior change and weight loss maintenance.

### Objectives

The overall aim of this study was to investigate *how* digital technology can meet end users’ values and needs by exploring and validating design features (ie, the combination and implementation of PSD principles and BCTs) through iterative design, development, and formative evaluation of a digital intervention called eCHANGE.

The following describes how 2 theoretical frameworks [52,55] targeting motivation and behavior change can be combined with applied innovation methodologies, such as design thinking [41,65] and Agile development [42], during the design of digital behavior change interventions. The findings show the development of digital technology aimed at facilitating sustainable behavior change to maintain weight after weight loss. The main research question of this study was as follows: how can an evidence-informed digital behavior change intervention, combining and implementing PSD principles and BCTs into design features, be designed and developed to support end user values and needs for long-term weight loss maintenance?

**Figure 1:**
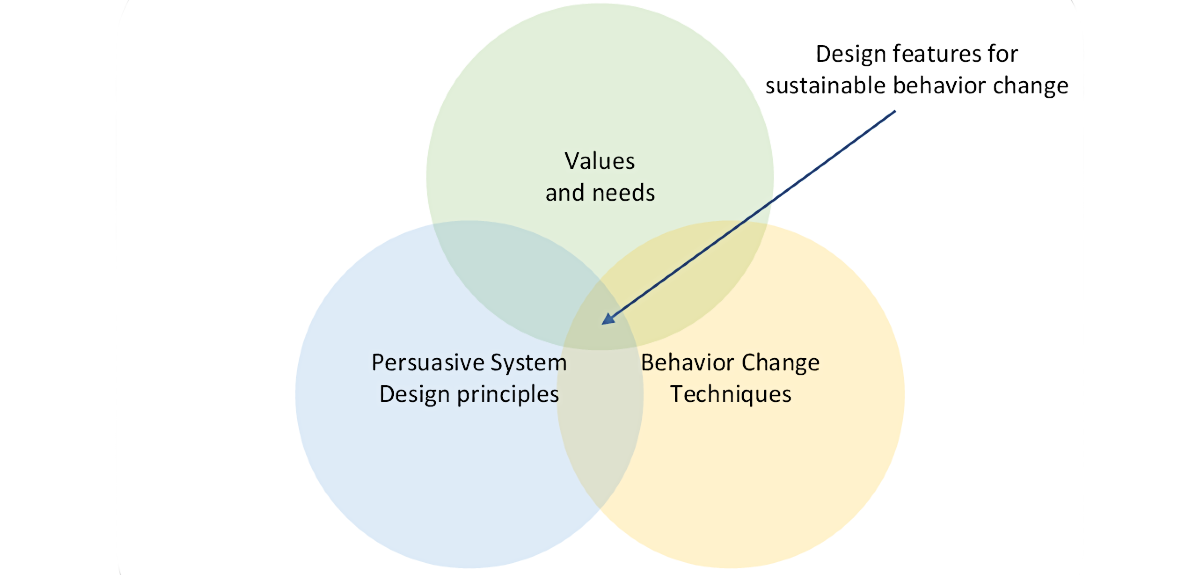
Design features for sustainable behavior change.

For this study, design features for (sustainable) behavior change were defined as the combination of *PSD* principles and *BCTs* embedded in a digital intervention, with the specific aim of supporting end users’ (ie, target users) values and needs to facilitate sustainable health behavior change (Figure 1).

## Methods

### The Double Diamond Framework and the Center for eHealth Research and Disease Management Roadmap

**Figure 2:**
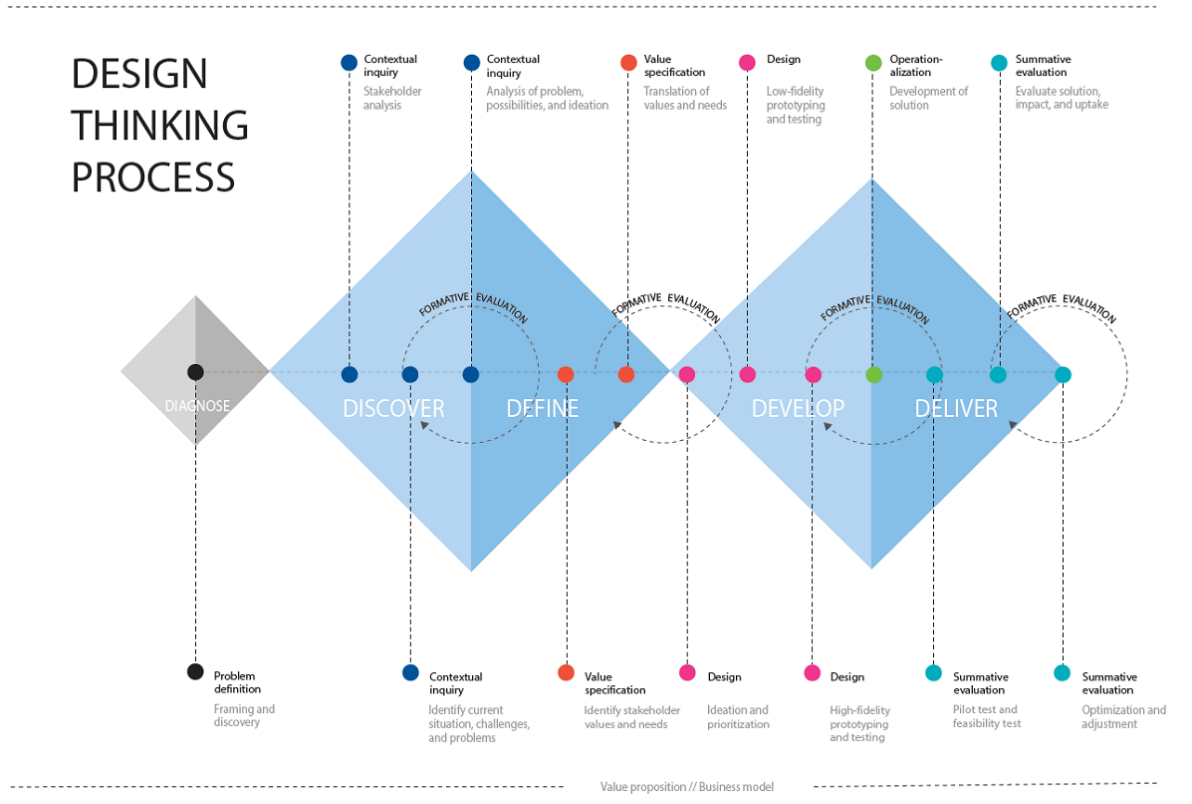
The Double Diamond [66] and the Center for eHealth Research and Disease Management Roadmap [36] combined: a design thinking process for eHealth design and development.

To develop and deliver a digital intervention aimed at supporting long-term weight maintenance following weight loss (ie, eCHANGE; ClinicalTrials.gov NCT04537988), the process was guided by the Double Diamond (ie, design thinking process) [65,66] and the Center for eHealth Research and Disease Management (CeHRes) roadmap [29,36] (Figure 2 and Multimedia Appendix 1 [36,66]). In addition, refer to the study by Asbjørnsen et al [32] for additional background and details. To improve the uptake and impact of digital technology [29], multidisciplinary collaboration, human-centered design approaches such as design thinking and service design [41,46], Agile software development principles [42], and formative evaluation were integrated into a systematic and iterative development process to optimize the fit between values and needs, technology, and context [36].

This study focused mainly on the second diamond of the Double Diamond process (ie, *develop* and *deliver*; Figure 2) [66], particularly the *Design* and *Operationalization* phases of the CeHRes Roadmap, including *formative evaluation* to gather input to improve the intervention [29,36].

During the *Design* phase [36], the aim was to explore how digital technology can be *developed* and to validate which design features meet end users’ values and needs to support long-term weight loss maintenance. Knowledge, insights, and ideas from previous studies [32,35] were translated into prototypes through co-design workshops [41], rapid prototyping [46], and user testing [29] with end users and other key stakeholders. PSD principles from the PSD model [55] and BCTs from the Cross-Domaine Taxonomy (version 1) by Michie et al [52] were combined and implemented into design features during several iterative cycles, providing ongoing information on how to address end user values and needs [32] (Figure 1).

The *Operationalization* phase [36] included Agile software development (ie, Scrum development sprints) [42] and testing of the digital intervention and focused on how the technology could be *delivered* and further improved to prepare for a feasibility pilot trial [29]. The PSD model [55] includes 28 individual PSD principles, which are categorized into 4 categories: *primary task support*, *dialogue support*, *system credibility support*, and *social support*. In comparison, the Cross-Domaine Taxonomy by Michie et al [52] comprises 93 distinct BCTs divided into 16 theory-independent clusters. The 2 frameworks applied to target behavior change [52,55] can facilitate standardized reporting of the PSD principles and BCTs embedded in a digital behavior change intervention.

In line with the iterative approach to eHealth development [36] and the Medical Research Council guidance for developing and evaluating complex interventions [54], the data collection, analysis, and results were intertwined throughout the design and development processes (Figure 2).

### Multidisciplinary Research and Development Team

Digital intervention development involved a multidisciplinary research and development team and was led by the study principal investigator (PI; LSN), a clinical psychologist with health psychology specialization and long-standing experience with digital behavioral interventions. The multidisciplinary team (14/45, 31%) entailed key stakeholders identified through stakeholder analysis [32] with diverse professional backgrounds and expertise, including researchers and clinicians in obesity and weight management, behavioral science, and eHealth; content editors; a digital designer; and software developers. In addition, a service designer was involved in facilitating service design workshops. Table 1 provides an overview of the multidisciplinary research and development team members, including their expertise.

**Table 1:** Overview of the multidisciplinary research and development team background and expertise (N=14).

Grouping	Total number, n	Obesity expertise, n	eHealth expertise, n	Behavioral and clinical health psychology expertise, n	Licensed health care providers^a^, n
Health care researchers	6	2	4	2	6
Content editors^b^	3	0	3	0	2
Design and software team	5	0	5	0	0

^a^For example, nurses, medical physicians, health psychologists, and physical therapists.

^b^1 content editor acted as product owner during the development phase.

### Recruitment of Study Participants: End Users and Other Key Stakeholders

End users were defined as people aged ≥18 years with a BMI of ≥30 kg/m^2^ [67] before weight loss (ie, who had lost ≥8% of their body weight through a low-calorie diet or behavior change program) who were in need of support to prevent weight regain. People who met these criteria and were able to speak and read Norwegian were invited to participate in this study. Recruitment was conducted at 3 secondary or tertiary obesity research and treatment centers (ie, hospitals) in Norway through convenience sampling. In addition, end users who participated in a prior formative study [32] and a group of healthy volunteers were invited to participate in usability testing. To compensate for time spent and potential costs (eg, parking and transport), the study participants (ie, end users and healthy volunteers) received a gift certificate (ie, approximately US $25 and US $50 for individual testing and workshops, respectively).

Representatives of other key stakeholders identified during stakeholder analysis [32] (eg, health care providers and behavior change and obesity experts) were recruited based on convenience sampling through the collaborating obesity research and treatment centers.

### Ethics Approval and Informed Consent

This study was approved by the Hospital Privacy and Security Protection Committee (ie, institutional review board equivalent; approval number 2017/12702) at the Oslo University Hospital in Norway. All study participants (ie, end users and other key stakeholders) received written and oral study information and signed an informed consent form before participation.

### Design and Development of the Digital Intervention

**Figure 3:**
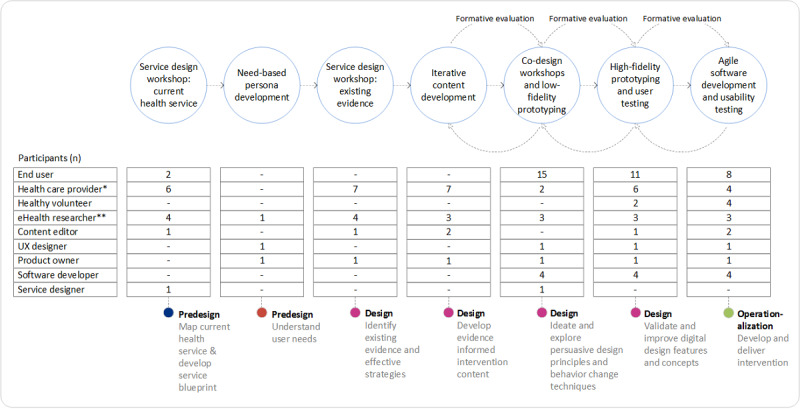
Overview of the iterative development process of the eCHANGE intervention, including activity participation (ie, n is the number of participants), based on the Double Diamond Approach. See Asbjørnsen et al [32,35] for the previous research and development steps. Some participants (ie, 4 end users and 3 health care providers) participated in >1 activity.*Health care provider (eg, clinicians and researchers/experts in obesity management: medical doctor, clinical dietitian, exercise physiologist, physical therapist, geneticist, psychologist). **eHealth researcher (eg, registered nurse, clinical health psychologist, health scientist, specialist health education and promotion).

The development process started with 2 predesign activities to prepare for the *Design* and *Operationalization* phases to *develop* and *deliver* the digital behavior change intervention. An overview of the design and development cycles during the eCHANGE intervention, including activity participation, is shown in Figure 3.

#### Predesign Phase

##### Service Design Workshop Mapping Current Health Services

First, a service design workshop [41] was conducted with user representatives (2/45, 4%) and other key stakeholders (11/45, 24%; ie, health care providers, eHealth researchers, and content editors) to gain insights into the current health services offered and to optimize the fit between the context, needs, and technology to be developed. The first author (RAA; a researcher and eHealth expert) and a service designer served as workshop facilitators. To develop a service blueprint [68], visualizing the current health services from a user perspective (ie, including user needs and experiences), the workshop (3 hours) focused on mapping the current health services and user journey (ie, with sticky notes on a large whiteboard).

##### Need-Based Persona Development

To create an image of future end users and facilitate an understanding of their needs and challenges, need-based personas (ie, user profiles) reflecting a subset of the identified end user values and needs [32] were developed by a digital designer, product owner (MO; ie, Scrum) [42], and the first author (RAA), inspired by existing guidelines [69,70]. A total of 6 personas were created reflecting the target group aspects and containing information related to demographics, weight history, social and health-related factors, habits, and common everyday challenges and needs (Multimedia Appendix 2). The personas were used as a design tool to ideate, create, and reflect on prototypes during the design process, explore how the solution could be developed, and ensure that some of the identified key end user values and needs [32] were considered.

#### Design Phase

##### Service Design Workshop Related to Existing Evidence and Content Development

A second service design workshop [41] (4 hours) was performed with other key stakeholders (10/45, 22%; ie, health care providers and eHealth, obesity, and behavior change experts). The aim was to identify evidence-based strategies associated with successful weight loss maintenance, including but not limited to existing research from, for example, the US National Weight Control Registry, determinants of weight loss maintenance [12,71-74], national [75,76] and international guidelines for a healthy lifestyle and obesity management [77-80], and stakeholders’ knowledge and experiences from weight management interventions. Relevant topics and themes to include in the content development to meet end user informational and educational support needs [32] were identified and facilitated (ie, using sticky notes and a whiteboard) by the first author (RAA) and a content editor.

Health care providers and researchers (10/45, 22%) from a wide range of disciplines with expert knowledge of obesity and weight management (eg, dietetics and nutrition, physical activity, physiology, medicine, and health psychology) contributed to content development (ie, educational material and skills training). The experts were also consulted throughout the technology development process to secure high-quality, evidence-informed intervention content based on existing evidence and BCTs known to be effective and promising in supporting weight loss maintenance [32,35].

As indicated in Figure 3, the content development process underwent several iterations based on end user feedback during the design process. Content development was led by the PI (LSN), assisted by content editors, who were responsible for editing and optimizing all written material (ie, texts, including images, videos, and voice-to-texts) during the digital intervention development. The aim was to enhance the value of the tailored information, feedback messages, educational material, and skills training included in the intervention. This was done through close collaboration between multidisciplinary experts and the research team, coordinated and adapted by editors, to create positive and meaningful intervention content, grab end user attention, and motivate change. The language used (eg, readability, clarity, and tone of voice) was given particular attention during the content development process.

##### Co-design Workshops and Low-Fidelity Prototyping

A total of 4 co-design workshops [41] (ie, research and design cycles, 3 hours each) with end users (15/45, 33%) were organized to ideate and explore how to meet end user values and needs to successfully maintain weight loss in the long term [32]. The prototyping comprised small iterative steps to obtain information on how to combine and implement PSD principles and BCTs. Low-fidelity prototypes (ie, paper prototypes and simple Marvel sketches) [29,46] were developed by the digital designer, together with participants from the research and development team, based on the previously identified PSD principles, BCTs, high-level requirements, and suggested design features [32,35].

The co-design workshops, facilitated by a service designer and the first author (RAA), started with an ideation session [41] to stimulate creativity and develop ideas on how to support 3 of the identified key values (ie, happiness, social support, and motivation) [32]: “What makes you happy?” and “How can a Virtual Coach help you keep focus and stay motivated?”

Low-fidelity prototypes were used to explore and validate the following design features: (1) *personalized self-monitoring*, (2) *goal setting and planning*, (3) *smart feedback* (eg, praise, rewards, reminders, and suggestions), and (4) *shaping knowledge* (eg, education and skills training) through various human-centered design methods (Table 2 and Multimedia Appendix 3). The participants were encouraged to share, sketch, and reflect on ideas and paper prototypes during the co-design workshops. Sticky notes and participant drawings were used to collect user feedback on how to create an engaging and motivating self-management technology, supporting healthy behaviors and weight loss maintenance.

**Table 2:** Formative evaluation: detailed overview of methods in the *Design* and *Operationalization* phase.

Formative evaluation methods and procedures		Design and operationalization		
		Co-design and low-fidelity prototyping^a^	High-fidelity prototyping^b^	Agile software development^b^
**A/B testing [43]**				
	Two versions of the design features (eg, horizontal vs vertical weight graph in relation to habits), were created, tested, and evaluated during to evaluate users’ preferences and validate features/concepts	✓^c^	✓	
**Expert reviews [29]**				
	Operationalization and combination of PSD^d^ principles [55] and BCTs^e^ [52]	✓	✓	
	Compliance with requirements for universal design, data protection by design and by default, and security guidelines (eg, web Content Accessibility Guidelines 2.0) [81-83]	✓	✓	✓
**Scenario based tasks [29]**				
	Four specific scenarios and tasks: animated onboarding and goal setting, creating a Week Plan, personalization of the intervention, and selecting favorite knowledge and skills training; after evaluating, if tasks could be successfully completed, the facilitator asked questions about the user experience		✓	
**Think-aloud technique [29]**				
	The participant could test the solution as they wished while sharing (ie, think aloud) what they did and why, accompanied by open-ended questions by the facilitator	✓	✓	✓
**The Sauro System Usability Scale [84]**				
	A brief questionnaire about system usability with a 1 (strongly disagree) to 5 (strongly agree) Likert scale was performed when the participant was alone in the room		✓	✓

^a^Workshops facilitated by a service designer and/or first author.

^b^Individual sessions facilitated by the Scrum product owner.

^c^Indicates which formative evaluation methods were applied.

^d^PSD: persuasive system design.

^e^BCT: behavior change technique.

##### High-Fidelity Prototyping and Usability Testing

During the next steps (Figure 3), the aim was to validate and improve the design features and intervention content at a high-fidelity level [29] during individual prototyping and usability testing sessions (45 minutes). A digital prototype of the intervention, developed using a web-based tool (ie, Marvel design platform), was presented on a smartphone to give a real-world look and feel and provide a certain degree of interaction (ie, gradually more realistic).

As presented in Table 2, a variety of formative evaluation methods were applied in combination to evaluate the high-fidelity prototypes and perform usability testing during the *Design* and *Operationalization* phases. The individual sessions with end users (12/45, 27%), health care providers (9/45, 20%), and healthy volunteers (4/45, 9%), facilitated by the product owner (MO), were voice/video recorded, whereas an observer (ie, digital designer, first author [RAA], or eHealth expert/content editor) collected notes. Some participants participated in >1 design and development cycle. The observer created a report with feedback and suggestions related to the design, content, and functionality, as well as additional input (eg, recurrent problems for, barriers to, and facilitators of use).

Stakeholders from the multidisciplinary research and development team (ie, eHealth researcher, digital designer, and software developers) contributed to prototype development based on participant feedback, focusing on graphical and conceptual design and performing expert reviews during the design and development process. Table 2 provides an overview of the formative evaluation methods applied, and Multimedia Appendix 3 includes additional details about participants involved.

#### Operationalization Phase

##### Agile Software Development

The *Operationalization* phase centered around the operationalization of design concepts through short development cycles based on Agile and test-driven development principles [43] to deliver a minimal viable product (MVP) for pilot testing in the real world (ie, ClinicalTrials.gov NCT04537988). The technology was developed during four incremental development sprints (ie, Agile software development and Scrum) [85] and is divided into (1) *habit tracking and smart feedback* (including rewards; ie, *Week Plan*), (2) *registrations and self-monitoring* (ie, *My Overview*), (3) *virtual coach and tips* (ie, *Virtual Coach and Smart, Tailored Feedback*), and (4) *animated effects, knowledge, and strategies* (ie, *Knowledge and Skills Training*). To optimize the efficiency of the development process, some of the iterative design and development cycles (ie, sprints) ran simultaneously.

To secure flexibility and quick adaptation of features based on the feedback collected during continuous validation and usability testing, the development team deployed a web-based software collaboration tool (ie, on the GitHub development platform) and daily stand-up meetings (ie, based on the Scrum methodology) [85]. The high-fidelity prototypes (ie, in Marvel), together with accessibility guidelines, personas, specified user stories (ie, functional requirements), and technical stories (ie, nonfunctional requirements), served as bases for the software development process. The user stories reflected the user needs and requirements for a desired feature and were intended to help the development team understand end user needs in relation to the system and its context. The user stories were written in an informal way from the user’s perspective: “As a [description of user], I want [functionality], so that [benefit].” Examples of user stories included the following: “as a user, I want to create my own plan, so that I can choose which habits I want to work on” or “as a user, I want to receive reminders, so that I do not forget to work on my habits.”

To maximize value and set the direction for software development (ie, Agile Scrum team), the product owner (MO) ensured that the product backlog was up to date (ie, prioritized list of requirements and acceptance criteria) and secured, along with the design and development team, rapid delivery of implemented features, and high-quality software based on user needs and requirements.

### Data Analysis

During the iterative intervention development (Figure 3), data collection and analysis provided ongoing information on how to improve the technology (eg, intervention content and operationalization of the design features). The prototypes evolved during co-design, rapid prototyping, and usability testing, where findings from one design cycle served as input to the next and set the direction for creation and validation of design features.

Rapid analysis [86] was applied during the design and development process to ensure that the collected data provided quick and thorough input to optimize the digital intervention (eg, actionable suggestions and specification of requirements through user stories). This included the structuring and summarizing of notes, including illustrative quotes and voice/video recordings from usability testing, into themes related to the design features to elicit input on how to combine and implement the PSD principles [55] and BCTs [52] into design features to support end user needs and preferences. In addition, a more in-depth directed content analysis [87] was performed to secure evidence-informed development and ensure that no themes were missed. This included the coding of study participants’ feedback and suggestions into predefined categories with respect to design, content, and functionality. The feedback and findings, including inconsistencies and conflicting needs, were first reviewed and discussed by the PI (LSN), 2 researchers (RAA and MLS), the product owner (MO), the digital designer, and/or a content editor to validate that the intervention content and design features (ie, PSD principles and BCTs) matched the identified end user values and needs. Thereafter, the findings were discussed (until consensus was reached) with participants from the multidisciplinary research and development team for continuous evaluation, prioritization, and improvement of features before the next iteration was conducted.

The product backlog reflected the prioritizations and decisions made by the research and development team. This was done to ensure that the selected design features and development of the intervention (ie, MVP) were in line with end user needs and preferences and that findings from previous research [32,35], evidence-informed knowledge, and feasibility considerations were taken into account before technical adaptations and updates were executed.

### Technical Architecture

The smartphone-based eCHANGE intervention app was designed and distributed as a native app through official app stores for iOS and Android. Web technology in a Cordova container was used, and when in use, all information is stored locally and encrypted with the Advanced Encryption Standard algorithm in Galois/counter mode before being written to the local device file system.

The first time the app runs, a 256-bit encryption key is generated. Between app invocations, an Advanced Encryption Standard key-wrapped algorithm with a (wrapping) key acquired from the user’s personal identification number is used and stored on the device keychain (ie, based on an existing technology platform) [88]. The keychain ensures that the mobile device is protected with a 4-digit personal identification number code or optional biometric authentication (ie, face recognition or fingerprint).

When in use, log data (ie, system use, including navigation, frequency of use, and use of functionalities) and self-monitoring data are sent through an encrypted channel to a secure server (ie, Services for Sensitive Data, University of Oslo) for future analysis and summative evaluation. If interested, the user may elect to import relevant personal data (eg, number of steps taken per day and weight measurements) from Apple Health or Google Fit on their device; however, this integration is neither required by the research team nor necessary to realize the full potential of the app.

### Security and Privacy Considerations

The eCHANGE intervention was developed by the Department of Digital Health Research at Oslo University Hospital in Norway in line with the national and international privacy and security standards and regulations (eg, Norwegian Digitalization Agency and European General Data Protection Regulations of 2018) [81-83]. A legal and privacy declaration was included as part of the information about the app in the settings functions in accordance with the existing requirements. The procedures applied in relation to the data protection impact assessment and risk assessment analysis of the technical solution were approved by the institution’s Department of Information Safety (approved in June 2020).

## Results

### Overview

This section focuses on the design operationalization of the incorporated PSD principles and BCTs into design features and main intervention components to address the following research question: *how can an evidence-informed digital behavioral change intervention, combining and implementing PSD principles and BCTs into design features, be designed and developed to support end user values and needs for long-term weight loss maintenance*?

To meet the diverse and dynamically changing needs of end users (ie, people aiming to maintain weight after weight loss) [32], the eCHANGE intervention was developed as a personalized self-management intervention and delivered as a smartphone-based app for flexible and easily available weight loss maintenance support.

### Participants

A total of 45 end users and other key stakeholders participated in intervention development, including 13 (29%) external stakeholders (ie, n=9, health care providers, including 1 health care manager and 4 healthy volunteers), 17 (38%) prospective end users, 14 (31%) stakeholders from the multidisciplinary research and development team, and 1 (2%) service designer. As presented in Multimedia Appendix 4, most end users were female (12/17, 71%), and the median age was 48 (range 30-63) years.

### Combining and Implementing PSD Principles and BCTs Into Design Features to Support End User Values and Needs for Long-term Weight Loss Maintenance

#### Identified Design Features

The various research and development activities performed in the study explored how a digital intervention can support identified key end user values (ie, *personalized care, feel supported, positive self-image, health, happiness, motivation, autonomy,* and *self-management)* [32] to maintain weight after weight loss. The study co-design and prototype validation resulted in the selection of 17 design features for (sustainable) behavior change to be included in the digital intervention (MVP). Further details are provided in Table 3.

The design features and main components of the digital intervention described in the following sections provide insight into how PSD principles and BCTs were combined and implemented to support end user values and needs for long-term weight loss maintenance.

**Table 3:** Identified design features and main components to support key end user values for long-term weight loss maintenance.

Main components and design features			Key end user values [32]														
			V1^a^		V2^b^		V3^c^		V4^d^		V5^e^		V6^f^		V7^g^		V8^h^
(A) Animated onboarding			✓^i^												✓		✓
**Week Plan^j^**																	
	(B) Behavioral planning and goal setting (eg, action and coping planning)	✓		✓								✓		✓		✓	
	(C) Motivational exercise and realistic goal setting			✓		✓						✓		✓		✓	
	(D) Habit rehearsal and tracking	✓		✓		✓								✓		✓	
**My Overview**																	
	(E) Personalized self-monitoring	✓		✓		✓		✓		✓		✓		✓		✓	
	(F) Goal setting target outcome	✓										✓		✓		✓	
	(G) Automatic integration of data	✓						✓		✓		✓				✓	
	(H) Visualization of target behavior in relation to target outcome	✓		✓		✓		✓		✓		✓		✓		✓	
**Knowledge and Skills**																	
	(I) Educational material and information	✓		✓		✓		✓		✓		✓		✓		✓	
	(J) Cognitive and motivational exercises	✓		✓		✓		✓		✓		✓		✓		✓	
	(K) My favorites	✓										✓		✓		✓	
**Virtual Coach and Smart Feedback System**																	
	(L) Virtual coach	✓		✓		✓		✓		✓		✓		✓		✓	
	(M) Animated nudging elements			✓						✓		✓					
	(N) Praise	✓		✓		✓		✓		✓		✓		✓		✓	
	(O) Rewards	✓				✓				✓		✓					
	(P) Reminders	✓										✓				✓	
	(Q) Suggestions			✓				✓				✓		✓		✓	

^a^V1: personalized care.

^b^V2: feel supported.

^c^V3: positive self-image.

^d^V4: health.

^e^V5: happiness.

^f^V6: motivation.

^g^V7: autonomy.

^h^V8: self-management.

^i^Indicates the design features identified to support the values of end users aiming to maintain weight after weight loss.

^j^Indicates the main intervention components.

#### Design Features and Main Intervention Components

##### The eCHANGE Intervention

**Figure 4:**
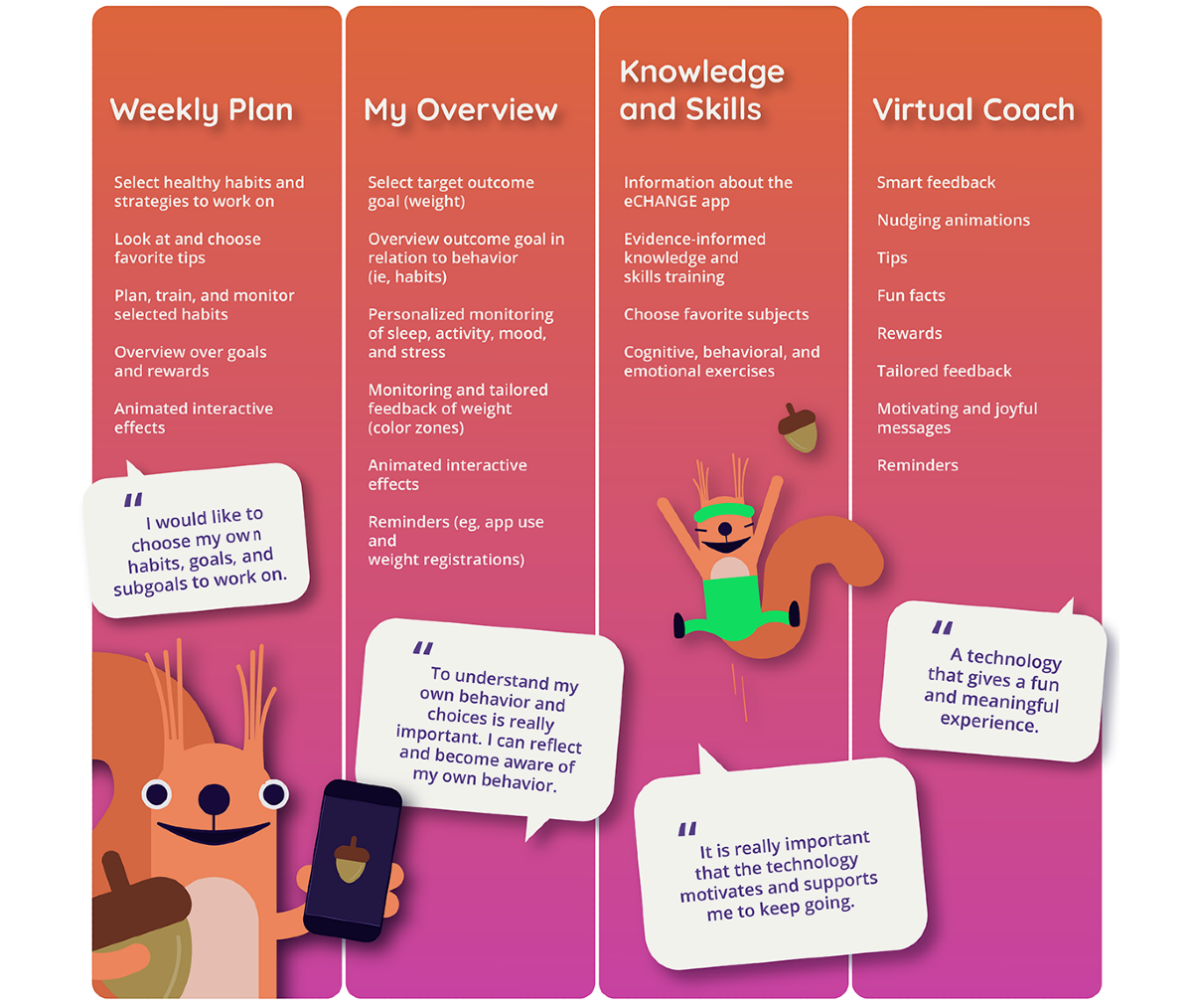
eCHANGE conceptual design and main intervention components to support end user values and needs.

The development of the eCHANGE intervention, incorporating the 17 design features presented in Table 3, resulted in an adaptive, interactive, and interconnected concept with four main components, as shown in Figure 4: (1) *Week Plan*, (2) *My Overview*, (3) *Knowledge and Skills*, and (4) *Virtual Coach and Smart, Tailored Feedback*.

On the basis of end user input wishing for a user-friendly, motivating, and personal intervention *that fits me*, an animated onboarding introduction (ie, design feature A, Table 3) was created to present the main features of the eCHANGE app. The onboarding feature could then be used to record baseline data (eg, current weight and weight maintenance goal) and tailor the intervention (eg, motivating messages and suggestions) to individual goals and needs. A general settings function for individual system preferences was also created to meet the end users’ individual preferences and needs for self-management support. This function evolved based on user and other key stakeholder feedback (ie, including usability experts from the multidisciplinary team) and included a range of personalization options (eg, frequency of reminders, type of feedback messages, automatic exchange of data, and dark or light mode).

##### Week Plan

During the co-design workshops and prototyping sessions, end users and health care providers emphasized the need for technology supporting planning and adherence to healthy lifestyle habits in their pursuit of maintaining weight and focusing on health and well-being (ie, not only weight). This included strategies on how to manage *high risk situations* (eg, situations with availability of tempting foods/snacks) and help with *impulse control* (eg, resistance to impulsive behavior such as comfort eating) to overcome lapses and prevent relapse into previous behaviors. One of the participants stated the following:

I would like to create a plan and choose which habits and goals to work on, kind of like a calendar.

End user

**Figure 5:**
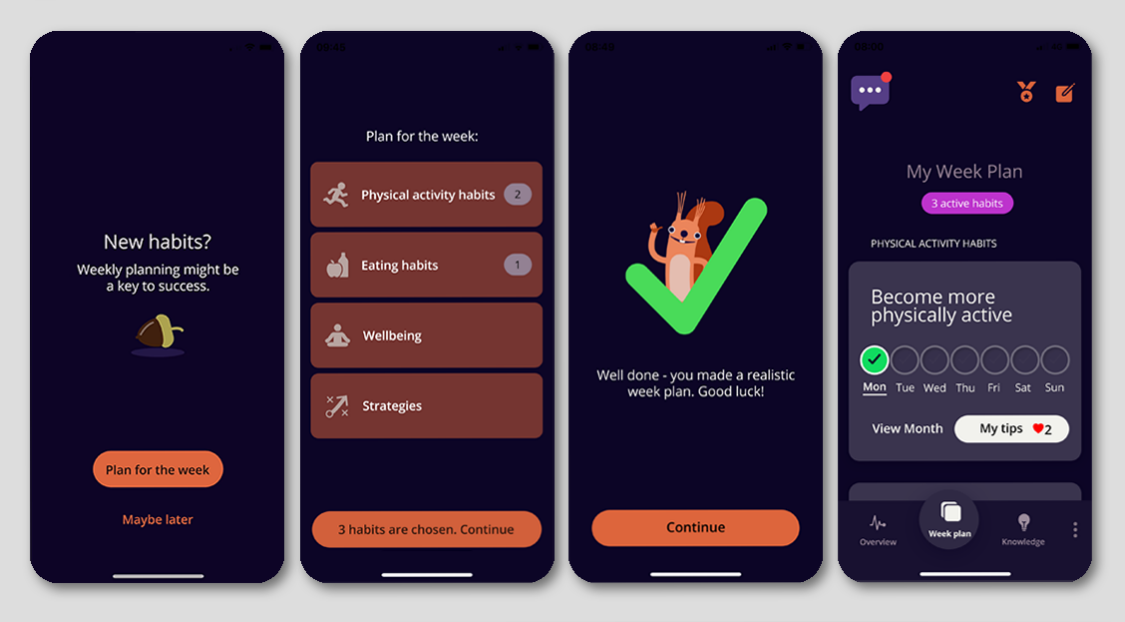
Screenshot of the eCHANGE program (ie, dark mode). Week Plan included the following design features: (B) behavioral planning, (C) motivational exercise and realistic goal setting, and (D) habit rehearsal and tracking.

A personal *Week Plan* was subsequently created to meet individual preferences and needs for healthy lifestyle changes to maintain weight. Figure 5 shows the eCHANGE *Week Plan* screenshot examples and included design features. The *Week Plan* contained options to select habits and strategies to work on from the following 4 categories: eating habits (eg, eating breakfast, planning meals, and healthy meals/snacking), physical activity habits (eg, daily walking goal), well-being (eg, sleep, stress management, and mindfulness), and self-regulation strategies (eg, problem solving, if-then plans, and back-on-track plan) (ie, design feature B, Figure 5), all associated with long-term weight loss maintenance [8,12,23,72,73].

Graded tasks (eg, subgoals or easy-to-reach targets) could be selected by the end user to facilitate self-efficacy and self-belief, as well as the adoption of physical activity and healthy eating habits. To support flexibility in planning, the *Week Plan* included an option for user-initiated changes whenever needed.

Information about health effects and *My favorite tips* (eg, introduction of environmental cues and restructuring of the physical environment) were also added to the habits and strategies presented in the *Week Plan*. This was done based on user feedback, aiming to provide knowledge and practical tips on how to adopt and maintain healthy habits as part of their daily routine. On the basis of suggestions from health care providers, a motivational exercise inspired by motivational interviewing techniques [89] was also incorporated to encourage realistic goal setting and stimulate motivation and self-efficacy during planning (design feature C, Figure 5). Building on previous formative results [32], as well as input from end users and eHealth experts, engaging and motivating design elements were added during the *Week Plan* design process. These design elements included tracking and monitoring of self-selected habits (ie, by checking the box in the personal plan; design feature D, Figure 5), animated prompts when tracking, weekly and monthly rewards related to personal targets, and positive feedback messages to stimulate motivation and adherence to healthy behaviors.

##### My Overview

Participating end users highlighted the need for an easy overview of the progress and performance of behavioral goals related to their weight maintenance goal, with the possibility of automatic integration of data (eg, from existing health apps and wearables). One of the participants stated the following:

I would like to have overview of my data in one place, to understand what works and does not work in order to maintain weight

End user

**Figure 6:**
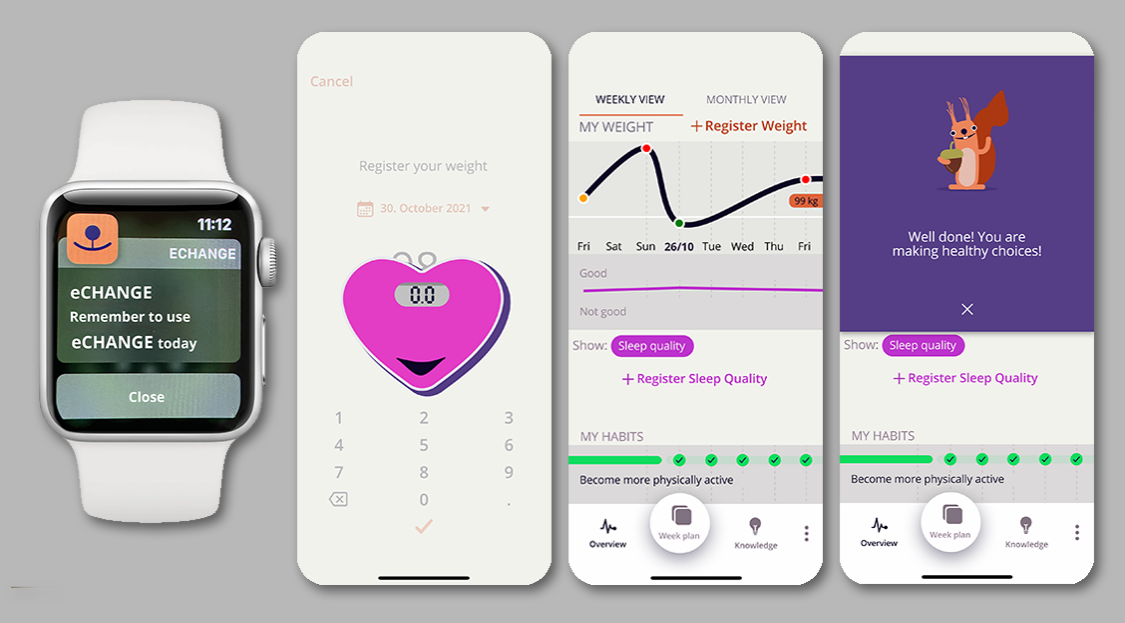
Screenshot eCHANGE program (ie, light mode). My Overview included the following design features: (E) personalized self-monitoring, (F) goal setting of target outcome, (G) automatic integration of data, and (H) visualization of target behavior in relation to target outcome.

Despite wishing for ways of monitoring progress, some end users expressed that they did not want to be *forced* by the system to register their weight. In contrast, health care providers emphasized the importance of daily or, at minimum, weekly self-monitoring of weight to encourage self-regulation and prevent regain. On the basis of this feedback, the component *My Overview* was created to support self-regulation and facilitate personalized self-monitoring. Figure 6 shows the eCHANGE *My Overview* screenshot examples and included design features.

In response to participant feedback, the possibility of automatically transferring data (ie, weight and steps) from existing health apps (ie, Apple Health and Google Fit) was included in *My Overview* to simplify self-monitoring and facilitate awareness and engagement. Health care providers (ie, obesity experts) also suggested a *traffic light* system [90] based on 3 color zones, as illustrated in Figure 6, design feature H, to provide visual and tailored feedback based on which *weight zone* users are in (ie, in relation to their target weight). The "green zone" was defined as <1.5 kg (ie, <3.3 lbs) above the target weight (eg, indicating to be "on track"), the "yellow zone" was when the weight increase was 1.5-3 kg (ie, 3.3-6.6 lbs) above target weight, and the "red zone" was an increase of >3 kg (ie, >6.6 lbs) above target weight.

In line with feedback from users not wanting to be forced to register weight, some end users also reported not wanting to focus on, or seeing, their body weight *all the time* and stated that they wished to be able to use the app in public spaces or show their progress to family and friends, without revealing their actual weight. Therefore, *My Overview* also included options for the user to choose when to register weight, whether to visualize actual weight or discrepancy from target weight, and a *hide the weight* option by clicking on their current weight. As end users highlighted holistic self-monitoring as an important feature, the possibility of monitoring self-selected habits, physical activity (ie, steps), stress, sleep, and mood over time was also incorporated in *My Overview*.

##### Knowledge and Skills

Several end users and health care providers in the study expressed the need for technology with trustworthy information about weight loss maintenance, including trustworthy (ie, evidence-based) information about strategies and skills to support and improve end users’ competence, autonomous motivation, self-regulation, and the ability to prevent weight regain. In response to this input, as well as previously identified end user values and informational support needs [32], a *Knowledge and Skills* component was created.

Content for this component was identified through end user and obesity specialist feedback. For example, the service design workshop with obesity management and behavior change experts identified 15 weight loss maintenance–related topics that were important to include in a weight loss maintenance–specific *Knowledge and Skills* section.

**Figure 7:**
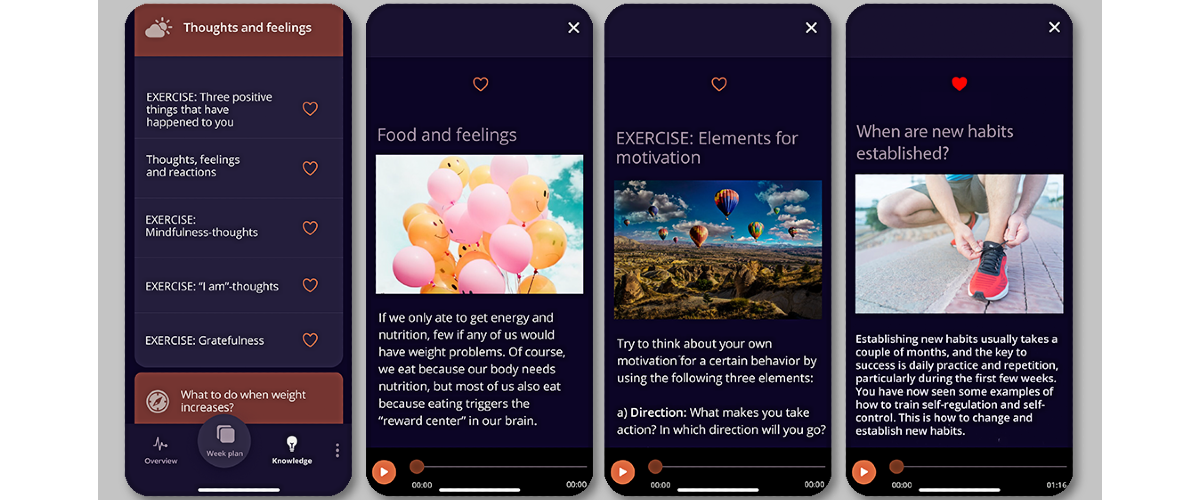
Screenshot eCHANGE program. Knowledge and Skills included the following design features: (I) educational material and information, (J) cognitive and motivational exercises, and (K) my favorites.

During the co-design sessions, end users also emphasized the need for educational material and information to be provided in an appropriate and understandable language, with brief textual information supported by images or videos and an audio option for listening rather than reading. This was implemented through iterative stakeholder testing. On the basis of input from end users, a *My favorite* option for the included information or exercises could also be chosen to facilitate easy access to relevant content based on individual preferences. Figure 7 shows eCHANGE *Knowledge and Skills* screenshot examples and the included design features.

In the co-design and prototyping sessions with end users and other key stakeholders, health and well-being, happiness, feeling of control and mastery, and motivation for long-term change were also identified. One of the participating obesity experts stated the following:

Many people lose faith in their capability to maintain weight

Obesity expert

To support autonomous motivation, self-belief, positive body image, self-efficacy, and self-regulation skills to prevent regain, experts on obesity and behavior change also identified 25 cognitive and motivational exercises to be included in the intervention. Theory-based exercises in the final eCHANGE intervention were anchored in BCTs identified as important to address sustainable behavior change to successfully maintain weight [32,35] and recognized motivational, self-regulation, and cognitive behavioral theories [89,91-96]. Table 4 provides an overview of the topics available in the eCHANGE *Knowledge and Skills* section.

**Table 4:** Overview over topics and content included in the eCHANGE *Knowledge and Skills* section.

Topic number	Topic	Content
1	Introduction	Introduction to the intervention program, main components, and general information about weight loss maintenance
2	Adaptive thermogenesis and energy balance	Information about body/physiological processes and challenges to maintain weight, including strategies to prevent weight regain
3	What is important to me?	Information and exploration of values; self-image, personal role models, identity, and thought patterns; value prioritization and life goals
4	How to change habits	About being in charge of own life, the nature of habits, awareness, behavioral patterns, and habit substitution; thoughts and behavior change and implementation of new habits
5	Becoming friends with the scale	Addresses the importance of self-monitoring and self-awareness for behavior change; thought patterns, positive self-talk, and self-confidence
6	Goal setting, planning, and problem solving	Defining realistic goals, regulation, and planning of healthy habits; relapse prevention and if-then plans and self-monitoring toward a personal goal
7	Motivation	Identity and values, internal drivers of behavior, types and factors of motivation, self-belief and behavior, thought patterns and self-belief, motivation, and relatedness
8	Food and drinks	Healthy diet and health effects; food and emotions/stress; healthy behaviors and health behavior change, awareness, habits and routines, nutrition, and healthy eating strategies
9	Physical activity	Physical activity and weight loss maintenance, barriers or physical challenges, strategies on how to incorporate physical activity into daily life, and training/exercise suggestions
10	Sleep	Information about circadian rhythm and sleep, importance of health, and quality of sleep; improvement of sleeping routines and health effects
11	Communication	Communication and your surroundings, body language, self-image, and positive self-talk
12	Social support	Types of social support and skills to strengthen social support systems; peer support; social and environmental cues for healthy and unhealthy habits and stimulus control
13	Thoughts, feelings, and stress	Relationship between thoughts and feelings, thoughts and stress, regulation of emotions and thoughts, thought reframing, self-efficacy, and positive self-image/self-esteem and body image
14	Weight maintenance and weight regain	Strategies for successful weight loss maintenance; traffic light system and weight zones; weight regain and causes; skills for self-regulation, problem solving, and relapse prevention
15	Mindfulness and relaxation	Introduction to mindfulness; practice self-compassion and strategies for stress management, including relaxation/mindfulness exercises to improve health and well-being

##### Virtual Coach and Smart Tailored Feedback

During the ideation and co-design sessions with end users, the need for positive, tailored, *just-in-time support* to reinforce healthy behaviors and motivation *to keep up and stay focused* was highlighted. This resulted in the co-design of a *virtual coach*, designed as an animated squirrel, as well as a *smart* feedback system developed to provide decision support and stimulate motivation and adherence to healthy behaviors. Although some end users expressed a wish to create their own personalized *virtual coach*, the essential factors, according to user feedback, involved content and expressions. One of the users stated the following:

It is not important how it (ie, the buddy) looks, but what it says and what it does.

End user

**Figure 8:**
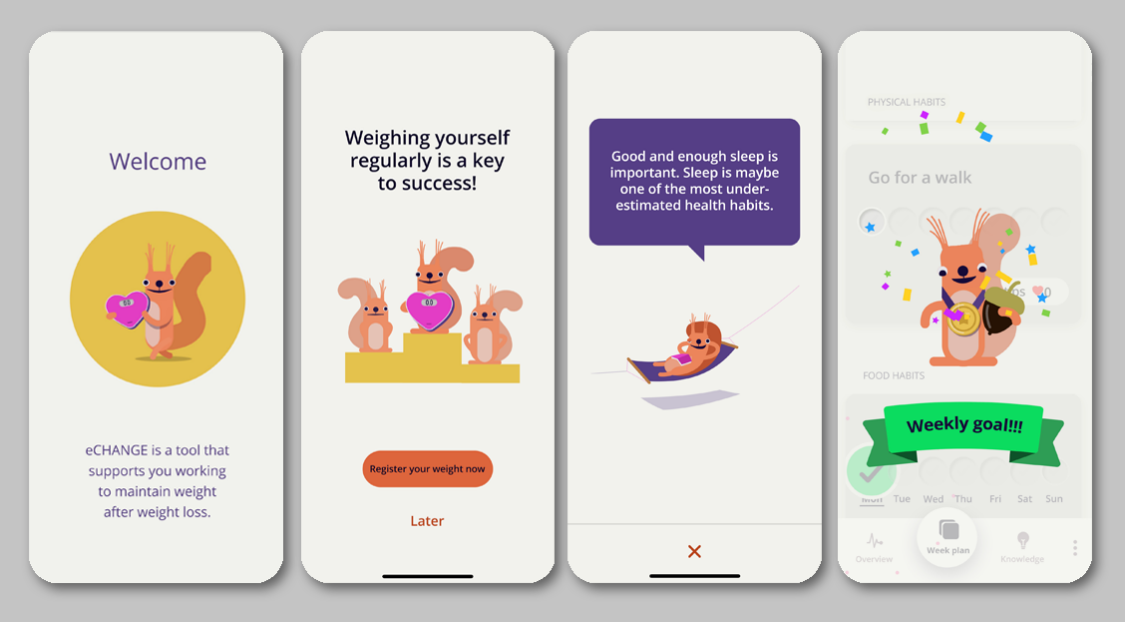
Screenshot eCHANGE program. Virtual Coach and Smart Tailored Feedback System included the following design features: (L) virtual coach, (M) animated nudging elements, (N) praise, (O) rewards, (P) reminders, and (Q) suggestions.

The *virtual coach* was co-created with end users and other key stakeholders to facilitate gradual engagement and guide the user through the first *setup* of a personal *Week Plan* and *My Overview*. Participating obesity and behavior change experts stated that the *virtual coach* should also provide feedback in line with professional coach advice. This led to the incorporation of feedback messages developed by health care professionals, with coaching techniques in accordance with motivational interviewing [89]. Figure 8 shows the eCHANGE *Virtual Coach* and *Smart Feedback System* screenshot examples and included design features.

To provide *just-in-time support*, the automated, smart feedback system was developed based on real-time self-monitoring data, dynamically adapting to the user based on a set of rules (ie, preference based and data-driven algorithms). The feedback system, developed by software experts in collaboration with obesity and behavior change experts, allowed tailoring of the intervention to individual progress. This included positive feedback on the performance of the behavior, close to target behavior; healthy lifestyle suggestions; information about health benefits of healthy lifestyle behaviors; suggestions to make healthy choices based on current weight zone (ie, green zone, yellow zone, and red zone); and help to get *back on track* (eg, when in the yellow or red weight zone). Multimedia Appendix 5 provides examples of feedback messages.

End users also reported preferring a *virtual coach* that would support habit formation through health-focused suggestions on how to maintain weight, prompt daily rehearsal of target behavior, and bring a sense of joy or happiness by suggesting new habits. Several animated nudging elements were integrated into the behavioral design during the high-fidelity prototyping and usability testing (Figure 8, design feature M) to support these preferences, promote healthy behaviors, and make the intervention desirable. When testing the intervention following this incorporation, one of the end users reported the following:

Opening the app puts me in a good mood

End user

The participating end users and health care personnel also suggested that the *virtual coach* should provide reminders that could stimulate adherence to the *Week Plan* and self-monitoring of weight, as well as weekly and monthly rewards to highlight goal achievement, as presented in Figure 8, design features O and P. In support of these suggestions, previous findings [32,35], and input from participating behavior change experts, encouragement to apply positive self-talk or choose self-selected rewards in line with individual values were incorporated to facilitate autonomous motivation and ongoing behavior change, without threatening autonomy.

#### PSD Principles and BCTs Implemented in the eCHANGE Intervention

To support weight loss maintenance values and the needs of end users, PSD principles from the 4 categories of the PSD model by Oinas-Kukkonen [55], such as *tailoring*, *personalization*, *self-monitoring*, *reminders*, *rewards*, *rehearsal*, *praise*, and *suggestions*, were implemented in the eCHANGE intervention. The PSD principles were combined with BCTs from 15 of the 16 BCT clusters from the Michie et al [52] taxonomy of behavior change techniques, such as *goals and planning*, *feedback and monitoring*, *social support*, *repetition and substitution*, *shaping knowledge*, *natural consequences*, *associations, antecedents, identity*, and *self-belief.*

The PSD principles of *personalization* and *tailoring* were incorporated into all main components to tailor the intervention to individual preferences and end user needs. For a description of the design features in the eCHANGE intervention and an overview of the design features, including PSD principles and BCTs combined and implemented, refer to Textbox 1 and Multimedia Appendix 6 [32,35,52,55]. Additional information about the formative design results from the system usability check (eg, System Usability Scale score) can be found in Multimedia Appendix 7 [84].

**Design features description to support sustainable behavior change and weight loss maintenance.**

**(A) Animated onboarding**

*Animated onboarding* introduces the app and guidance to create a personal *Week Plan* and *My Overview* to shape the future interaction (eg, how the content is delivered and visualized)

**(B) Behavioral planning and goal setting**

Supports creation of action and coping plans with self-selected healthy habits and goals, preplanning for potential barriers, and prevention of relapse (eg, weekend temptations and back-on-track strategies).

**(C) Motivational exercise and realistic goal setting**

*Motivational exercise* supports realistic goal setting and creation of a feasible *Week Plan*; the exercise (eg, self-evaluation bar 1-10) facilitates reflection and commitment to the plan

**(D) Habit rehearsal and tracking**

The *Week Plan* includes a tracking tool to follow up on self-selected healthy habits in a weekly or monthly overview (ie, calendar function), monitor progress, and support rehearsal and adherence to the plan

**(E) Personalized self-monitoring**

*Personalized self-monitoring* allows for registration of body weight, physical activity (ie, steps), perceived mood, stress, and sleep over time; visualized in a weekly or monthly overview; historical data is available

**(F) Goal setting target outcome**

*Goal setting* allows the user to set an outcome goal (ie, weight target/weight maintenance goal); actual weight or discrepancy from target weight (eg, +3 kg or –3 kg) is visualized in *My Overview*

**(G) Automatic integration of data**

*Automatic integration* of data is available for activity tracking (ie, steps) and body weight (ie, through Apple Health and Google Fit)

**(H) Visualization of target behavior in relation to target outcome**

A *personal visualization* of the target behavior(s) (ie, chosen habits) in relation to target outcome (ie, weight) over time in a weekly or monthly overview through graphs and icons in relation to weight zones (ie, green, yellow, and red); provides means for understanding the link between cause and effect of behavior and outcome to support awareness, self-reflection, and self-regulation; a progress bar (ie, goal gradient) related to each habit, visualization of goal progress and adherence to the plan and behavioral performance in relation to individual targets

**(I) Educational material and information**

*Educational material and information* through 15 topics related to sustainable weight loss maintenance and behavior change; provided through text or audio and videos

**(J) Cognitive and motivational exercises**

*Skill training* through 25 cognitive behavioral and motivational exercises to support skills related to behaviors, thoughts, and emotions and improve self-belief (eg, focus on past success and positive self-talk) and continued motivation for sustainable behavior change (eg, identity and personal values)

**(K) My favorites**

A general personalization feature where the user can mark and view only *My favorite* tips, skills training, knowledge, exercise, and/or strategies for easy access to personalized content and decision support

**(L) Virtual coach**

A *virtual coach* (ie, animated coach/buddy) provides automated, tailored (decision) support (ie, smart feedback—a data- and preference-driven algorithm enables smart feedback and tailoring of the intervention), including motivating messages, prompting of weight maintenance strategies, information about health effects, and self‐reward/self‐praise when reaching goals or performing target behavior. The virtual coach adopts a social, supportive role (eg, motivating interviewing techniques) and provides real-time progress/performance feedback related to health maintenance behaviors based on outcome data or weight zones (ie, green, yellow, or red zone in relation to target weight), physical activity data, habit tracking/behavior self-monitoring, and user data from the past 30 days; in the general settings function, type of feedback messages can be selected based on personal preferences

**(M) Animated nudging elements (eg, prompts/cues)**

*Animated elements* were provided to prompt, encourage, and positively reinforce healthy behaviors and decisions to reach target goals/desired behavior through enjoyable and surprising animated elements, such as a “heart scale” pop‐up to encourage weight registration, animated effects (ie, firework/sparks) to stimulate adherence, and animated prompts close to target behavior or to elicit/trigger healthy behaviors

**(N) Praise: positive feedback**

*Praise* is provided through positive, tailored feedback messages close to target behavior (ie, real time) when reaching individual goals to recognize efforts and success and, unexpectedly, to stimulate motivation to sustain a healthy lifestyle

**(O) Rewards**

*Rewards* (eg, medal and confetti) are provided when reaching self‐selected healthy habit targets to highlight goal achievement, facilitate engagement, and positively reinforce progress (eg, weekly/monthly reward)

**(P) Reminders**

*Reminders* through “pop‐up messages” on a mobile device/compatible smartwatch to facilitate engagement and adherence (eg, behavioral practice and weight registration); personal frequency and type of reminder choice

**(Q) Suggestions**

*Suggestions* provided by the virtual coach or through animated prompts/cues to support healthy lifestyle habits (eg, suggestions of healthy habits and practical strategies in everyday life to keep weight off)

## Discussion

### Principal Findings

This study provides insights into the design and development of a digital behavior change intervention called eCHANGE, which aims to combine and implement PSD principles and BCTs into design features to support end user values and needs for long-term weight loss maintenance. The results revealed specific design features for sustainable health behavior change to prevent weight regain, combining PSD principles and BCTs, as well as how these design features could be operationalized into core components during the design and development of the digital intervention.

### Combining and Implementing PSD Principles and BCTs Into Design Features to Support End User Values and Needs for Long-term Weight Loss Maintenance

On the basis of participant feedback, 17 design features were identified in this study (Table 3) to support 8 previously identified key end user values for weight loss maintenance [32]. During the co-design and prototype sessions, the values of *self-management*, *personalized care*, and *motivation* received the most attention and feedback from end users. The identified design features were implemented in the digital intervention through 4 interconnected main components: *Week Plan*; *My Overview*; *Knowledge and Skills*; and a *Virtual Coach and Smart, Tailored Feedback System*.

The findings indicate that to support the identified, interconnected end user values and needs [32], digital weight loss maintenance interventions should include design features that focus on health and well-being (ie, not only weight); facilitate the generation of habitual behavior, self-regulation, autonomous motivation, knowledge, and skills; and provide positive, tailored support to maintain weight after weight loss in the long term. Existing research has pointed toward a need for such an approach [32,35] and that a combination of PSD, BCTs, and behavior change theories might facilitate the design of effective technology-based tools and strategies for behavioral obesity interventions [35,58]. However, to the best of our knowledge, this is the first study to show how PSD principles and BCTs can be translated into design features to support end user values and needs to maintain weight in the long term. In line with existing research [33,97], the findings indicate a need for a shift in goal focus from *weight* to *health and well-being*. This may suggest that technologies for digital weight loss maintenance should aim to support healthy lifestyles and sustained motivation in line with the self-determination theory, supporting psychological needs such as competence, relatedness, and autonomy [26,92,95]. Digital interventions incorporating features supporting habit formation in line with self-determined goals, individual values, and identity, as well as focusing on meaningful areas such as improved health, might also enhance a sense of purpose and facilitate long-term health behavior changes and weight outcomes [17,26,32,98-101]. The results also indicate that the application of PSD principles from the *primary support*, *dialogue support*, *social support,* and *credibility support* categories might be required to aid weight loss maintenance and that the application of PSD principles such as *personalization* and *tailoring* of design features is important to match individual challenges, goals, and key values.

The eCHANGE intervention was developed as a personalized (adaptive) digital intervention aimed at providing self-management support for long-term weight maintenance. The results illustrate how PSD principles can be combined with BCTs that have been identified [32,35] as effective and promising in supporting weight loss maintenance and how they can be integrated into a digital intervention based on multidisciplinary stakeholder feedback. Existing research has indicated that BCT combinations targeting motivation and persistence in health-promoting interventions might increase the chances of successful health behavior change [102]. A systematic review identifying active ingredients in complex behavioral interventions for adults with obesity also indicated that the inclusion of BCTs in interventions could be beneficial, facilitating assorted phases of the behavior change process [53]. As such, the adaptive intervention format of eCHANGE can potentially support the behavior change required to maintain weight, allowing the user to choose between a number of BCTs depending on individual needs.

Development of healthy habits and adjustment or *breaking* of less healthy habits are required to optimize and maintain the health benefits of weight loss, as well as maintain new weight, over time [103,104]. Therefore, self-regulation strategies and skills are essential to enhance when new physically active lifestyles or healthy eating patterns are not fully automated and are likely vital to maintain healthy behaviors and not relapse into previous habits [26,105]. The findings from this study show that targeting holistic aspects (eg, cognitive, emotional, social, and behavioral) of behavior change [103] during the design of digital technologies may be essential when aiming to deal with the often multifaceted challenges and needs of weight loss maintenance [23,32,98]. Informed by the self-determination and self-regulation theories [91,92,95], some of the features identified in this study focus on the generation of habitual behaviors through recognized self-regulation and habit formation techniques. The application of habit-forming techniques could free cognitive capacity to facilitate engagement in desired behaviors that may help adopt health-related behaviors to maintain weight loss in the long term [104,106,107]. The incorporation of BCTs in digital interventions, such as self-regulatory strategies to bridge the intention-behavior gap (eg, *if-then plans/problem solving*)*,* habit-related techniques (eg, *graded tasks*), and self-belief (eg, *focus on past success, self-talk*), could also be important for targeting specific values (eg, self-management, motivation, and positive self-image) or mechanisms of action (eg, behavioral regulation and beliefs about capability) that may facilitate continued behavior change and weight gain prevention [15,25,32,104,108-112]. A literature review aimed at identifying links between BCTs and mechanisms of action [112] indicated that some of the core BCTs incorporated in the eCHANGE intervention could affect behavior change mechanisms (eg, *beliefs about capabilities, behavioral regulation,* and *motivation*), which is in line with identified weight loss maintenance values and needs [32]. This underlines the importance of keeping in mind the links among intervention content, design features, and the values and needs of the target group when designing and developing digital interventions for weight loss maintenance.

Human behavior is affected and shaped by a range of individual factors, including cognitive, psychological, biological, physical, and emotional factors; habits; values; motivational and demographic factors; and external factors such as environmental, cultural, social, and physical contexts in which behaviors occur [113]. Therefore, when designing digital technologies for sustainable behavior change, a fit between technological, human, and contextual factors is required [36,114]. In addition, the success or failure of digital interventions subsequently depends on whether individual end user needs are met [32,115]. The development of technology-supported programs for weight loss maintenance based on theory, evidence, and person-based approaches is gradually receiving increased attention [116-119]. The eCHANGE intervention’s value-based approach to eHealth development, in which PSD principles and BCTs identified to match end user values and needs [32] were gradually embedded into the digital technology through the design features, represents a major novelty. However, how persuasive and behavior change strategies are operationalized into design features may affect the usefulness and effectiveness of technology in terms of health outcome improvements. This points to potential issues, as the use of digital interventions that do not fit end user values and needs could affect intervention acceptance, adoption, and diffusion, indicating that a person-centered and iterative approach is needed [29,31,32,36].

A recent scoping review examining human-centered eHealth development indicated that because of the complexity of eHealth development, multiple strategies and methods should be combined in line with the research objectives when conducting such studies [115]. As such, a combination of theory, research-based, creative, and innovative methods, guided by the Double Diamond (ie, design thinking process) [65,66] and the CeHRes Roadmap [29,36] (Figure 2), can be applied to translate values and needs from ideation to the operationalization of design features. The application of co-design and Agile development methods during this process may increase the chance that the values and needs of (future) users are met [43,45,110]. The tailoring and personalization of digital technologies in line with the values of individuals might be a promising motivational strategy for continued health behavior change [32]. Therefore, the human-centered and value-driven approach can be useful in strengthening the self-regulatory capacity and autonomous motivation required to achieve sustainable behavior change [26,32,120]. The application of theory-based approaches [52,55] when developing complex digital behavior change interventions such as eCHANGE can also facilitate specification and transparency of the internal structure of the technology and may enable the development and evaluation of high-quality and effective digital behavior change interventions [29,35,121].

### Recommendations for Future Research

Future research should aim to investigate how digital technologies can be effective in facilitating sustainable behavior change and successful weight gain prevention and whether the identified design features, PSD principles, and BCTs contribute to long-term behavior change and sustainable health effects. In addition, while sustained technology engagement is not necessarily needed for a digital intervention to be useful, long-term weight loss maintenance supported by digital tools requires some degree of use. The degree of use needed and the design features that best support engagement and motivation for continued health behavior change and long-term weight maintenance are yet unknown. Therefore, future research should aim to evaluate technology engagement related to individual differences and the design features or intervention components that contribute to the creation of engaging technologies [122,123]. Hence, to optimize the impact of digital health interventions through personalized design, future research should also aim to provide more knowledge on how to tailor and personalize digital weight loss maintenance interventions (eg, design preferences) to support identified values and needs for long-term weight maintenance [32].

Finally, studies testing the actual use of the technology (eg, through log data analysis) may also provide knowledge related to end user preferences and needs (eg, latent, contextual, and future needs) and help identify which features may work best for whom and why to maintain weight. As such, a feasibility pilot trial may be a cost-effective way of acquiring more knowledge related to feasibility, desirability, and preliminary efficacy to support weight loss maintenance [54]. A feasibility pilot trial can also provide insights into user experiences and the actual use of the different components of technology to optimize and tailor digital interventions. Future research should also aim to evaluate the (long-term) effectiveness of weight loss maintenance and health-related outcomes through randomized controlled trials. To find the most effective combination of design features to support behavior change and weight loss maintenance over time, experimental designs, such as fractional factorial designs, could also be performed to test which features or combinations of features might work best for whom and when [29].

### Strengths and Limitations

This study had some limitations. First, a few participating end users were still aiming to reduce their weight, although their initial goal for weight loss had been achieved. This could be a limitation for this study as end user needs during weight loss may differ from needs when aiming for weight maintenance [32,35]. However, the balance between weight loss and weight regain likely reflects real-life aspects and challenges related to weight loss maintenance, and this balance was also reflected in the technology design (eg, availability of *back-on-track* features).

Second, with respect to end user participation, more women than men participated in the study. There may be gender differences in weight loss maintenance strategies; therefore, this gender imbalance may limit the generalizability of the intervention to men, although several other key stakeholders participating were men, which might strengthen a more representative perspective.

Third, the participants in this study reported having a high school education or higher, which could indicate that this was a fairly well-educated sample. As such, the sample may not fully represent the diversity of the future end user population (ie, people with obesity aiming for weight maintenance after weight loss). However, existing research demonstrates inconsistent patterns of engagement and nonuse/attrition with respect to user characteristics (eg, education, age, and gender) [124,125]. In addition, the reported expectations and perceptions of a certain technology might not predict its actual use in practice [29]. To address these possible limitations, data from this study, as well as a previous study [32], were used to create user profiles (eg, need-based personas and user stories) representing potential end users and to guide the co-design and implementation process of adaptive, persuasive features to tailor the technology to individual preferences.

Fourth, because of limited time, resources, and privacy and security restrictions, some end user/stakeholder wishes (eg, personalized virtual coach, context-aware suggestions, social learning, and collaboration with other users) were not reflected in the current MVP. On the basis of the nature of Agile development, the selected design features were prioritized with respect to practical (eg, privacy and security issues) and cost-effectiveness considerations (ie, value-based prioritization based on stakeholder input).

This study has numerous strengths. The focus on high multidisciplinary stakeholder involvement and end user values and needs are such strengths. Formative design results from co-design workshops, prototype validation, and usability testing with end users and other key stakeholders provided input with respect to what to deliver, as well as how, to support weight loss maintenance through design features (eg, content, function, and design). This highlights the importance of multidisciplinary collaboration between end users and other key stakeholders during the design and development of digital interventions to meet end user values and needs. Another strength is the focus on, and novelty of, how PSD principles and BCTs can be combined and implemented into design features of digital weight loss maintenance interventions. The application of the design features identified in this study could also be relevant for other areas requiring continued health behavior change, as the identified needs might reflect universal values or drivers for sustained behaviors [32].

### Conclusions

To the best of our knowledge, this study is the first to combine PSD principles and BCTs into design features during the design and development of an evidence-informed digital behavior change intervention to support end user values and needs for long-term weight loss maintenance. The findings indicate that personalized digital weight maintenance interventions should aim to support health and well-being by including design features and strategies supporting the self-regulation of behaviors, thoughts and emotions, habit formation, autonomous motivation, competence, skills, and tailored support. The design and development of the eCHANGE intervention can provide valuable input for future design and tailoring of evidence-informed digital interventions, even beyond digital interventions in support of health behavior change and long-term weight loss maintenance.

## Appendix

Multimedia Appendix 1 A design thinking process for eHealth design and development.

Multimedia Appendix 2 Description of the need-based personas.

Multimedia Appendix 3 Formative evaluation: detailed overview of methods and participants.

Multimedia Appendix 4 Overview of end user demographics.

Multimedia Appendix 5 Virtual Coach and Smart, Tailored Feedback system.

Multimedia Appendix 6 Overview of design features, persuasive system design principles, and behavior change techniques combined and implemented.

Multimedia Appendix 7 System Usability check.

## Abbreviations

BCT: behavior change technique
CeHRes: Center for eHealth Research and Disease Management
MVP: minimal viable product
OUH: Oslo University Hospital
PI: principal investigator
PSD: persuasive system design
VHT: Vestfold Hospital Trust
