# Supplementary figures and images for: Combining Persuasive System Design Principles and Behavior Change Techniques in Digital Interventions Supporting Long-term Weight Loss Maintenance: Design and Development of eCHANGE
Source: JMIR Hum Factors. 2022 May 27;9(2):e37372. doi: 10.2196/37372 (PMC9187967; doi:10.2196/37372)

# DESIGN THINKING PROCESS

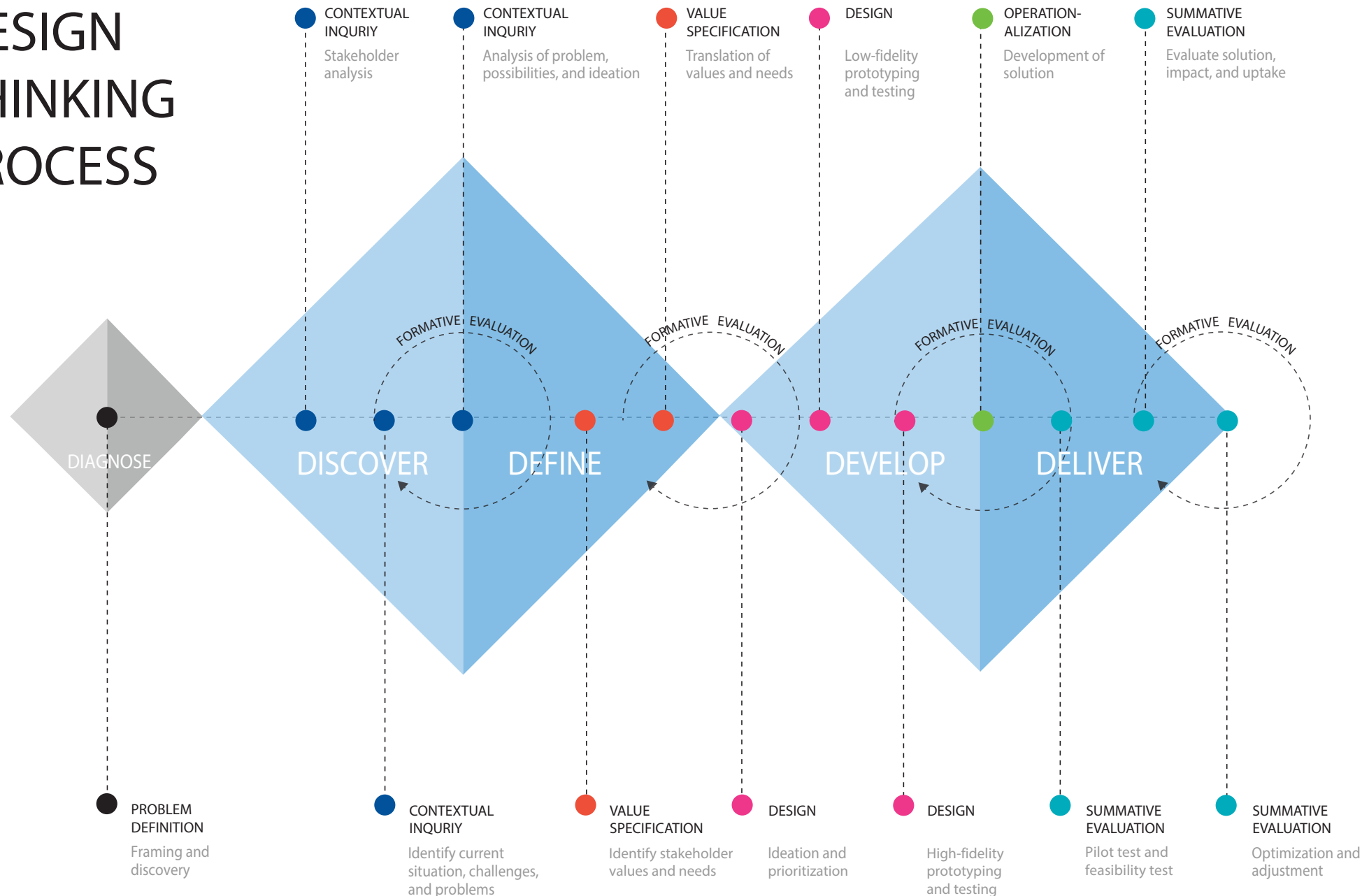

Supplement: Multimedia Appendix 1 [file humanfactors_v9i2e37372_app1.pdf]
