# Supplementary material for: Combining Persuasive System Design Principles and Behavior Change Techniques in Digital Interventions Supporting Long-term Weight Loss Maintenance: Design and Development of eCHANGE
Source: JMIR Hum Factors. 2022 May 27;9(2):e37372. doi: 10.2196/37372 (PMC9187967; doi:10.2196/37372)
Supplement: Multimedia Appendix 2 [file humanfactors_v9i2e37372_app2.pdf]

MULTIMEDIA APPENDIX 2

Design tool: Description of need-based personas main characteristics, challenges and needs.

Persona	Demographics	Challenges	Needs
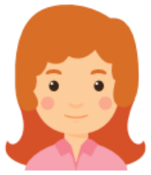 **Sana**	Woman 58 years	Sana experiences her work as stressful and feels tired, which makes it challenging to maintain weight.	Sana would like to understand her own behavior, what works and does not work to prevent weight regain. She also wants to find new ways to get more energy to be able to play with her grandchild, reduce stress, and keep up with her new, healthy routines to maintain weight.
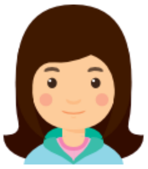 **Ylva**	Woman 47 years	Ylva has lost and gained weight many times. After several years being overweight, she has started to experience health problems.	Ylva is tired of registering everything she eats all the time, and would like to have a plan to work on healthy routines. A plan which she can also use for weekends and holidays, in addition to “crisis situations” when she feels hungry and “have to eat”.
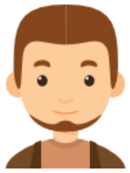 **Ben**	Man 53 years	Ben tries to be physically active and eat a healthy diet to maintain weight, but struggles to stay motivated.	Ben would like support to improve his health, prevent disease, and stay motivated. He just bought a smart watch for being active, and would like to be able to set his own targets and view his progress regarding weight and health.
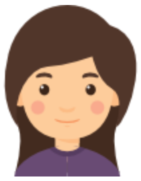 **Eva**	Woman 22 years	Eva has struggled with her weight since she was very young. She has finally lost weight, and does not want her new trousers to become too small.	Eva feels she needs a “boost” to keep her focus and motivation and continue with the healthy habits and exercising with her friends.<br>She sometimes feels that it takes a lot of effort to keep up with the healthy routines, and would like suggestions to maintain her weight and not fall back into “old habits”.
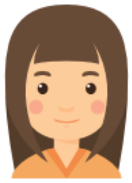 **Anna**	Woman 32 years	Anna was inside a lot of the time last year, as she had a baby. She misses having a social life and social contacts, but struggles with her confidence and body image.	Anna has just started a new job and looks forward to meet her new colleagues. Every Thursday there is a social gathering at work with coffee and chocolate. She feels guilty if she eats a chocolate, or does not show up. Anna would like to learn strategies on how to master these types of “risk situations”, and to regulate her thoughts and feelings.
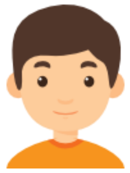 **Alexander**	Man 45 years	Alexander has some mental and physical health challenges related to his disease. He lives alone, and finds it difficult to prioritize his own health.	Alexander just lost weight; 34 kilos. He noticed that the unhealthy habits affect his sleep, how he feels and also how he experiences his body. Alexander would like to receive support in an inspiring way, and to maintain healthy lifestyle habits on “good and bad days” to prevent weight regain. He also would like to understand how the body works, and how to increase his activity level.
