# Supplementary material for: Combining Persuasive System Design Principles and Behavior Change Techniques in Digital Interventions Supporting Long-term Weight Loss Maintenance: Design and Development of eCHANGE
Source: JMIR Hum Factors. 2022 May 27;9(2):e37372. doi: 10.2196/37372 (PMC9187967; doi:10.2196/37372)
Supplement: Multimedia Appendix 3 [file humanfactors_v9i2e37372_app3.pdf]

**MULTIMEDIA APPENDIX 3**

Formative evaluation: detailed overview of methods and participants in the *Design* and *Operationalization* phase.

| Formative evaluation methods and procedures | Design and operationalization | | | Participants | | | | |
|---|---|---|---|---|---|---|---|---|
| | **Co-design and low-fidelity prototyping[a]** | **High-fidelity prototyping[b]** | **Agile software development[b]** | **End users** | **Health care providers** | **Healthy volunteers** | **eHealth researchers** | **Digital designer and software developers** |
| **A/B testing [43]** | | | | | | | | |
| Two versions of the design features (eg, horizontal vs vertical weight graph in relation to habits), were created, tested, and evaluated during to evaluate users' preferences and validate features/concepts | ✓[c] | ✓ | | ✓ | | | | |
| **Expert reviews [29]** | | | | | | | | |
| Operationalization and combination of PSD[d] principles [55] and BCTs[e] [52] | ✓ | ✓ | | | | | ✓ | |
| Compliance with requirements for universal design, data protection by design and by default, and security guidelines (eg, web Content Accessibility Guidelines 2.0) [81-83] | ✓ | ✓ | ✓ | | | | | ✓ |
| **Scenario based tasks [29]** | | | | | | | | |
| Four specific scenarios and tasks: animated onboarding and goal setting, creating a Week Plan, personalization of the intervention, and selecting favorite knowledge and skills training; after evaluating, if tasks could be successfully | | ✓ | | ✓ | ✓ | | | |

| | | | | | | | | | |
|---|---|---|---|---|---|---|---|---|---|
| | completed, the facilitator asked questions about the user experience | | | | | | | | |
| **Think-aloud technique [29]** | | | | | | | | | |
| | The participant could test the solution as they wished while sharing (ie, think aloud) what they did and why, accompanied by open-ended questions by the facilitator | ✓ | ✓ | ✓ | ✓ | ✓ | ✓ | | |
| **The Sauro System Usability Scale [84]** | | | | | | | | | |
| | A brief questionnaire about system usability with a 1 (strongly disagree) to 5 (strongly agree) Likert scale was performed when the participant was alone in the room | | ✓ | ✓ | ✓ | ✓ | ✓ | | |

[a]Workshops facilitated by a service designer and/or first author.
[b]Individual sessions facilitated by the Scrum product owner.
[c]Indicates which formative evaluation methods were applied and participants involved.
[d]PSD: persuasive system design.
[e]BCT: behavior change technique.


**References:**

29. L. van Gemert-Pijnen SMK, H. Kip, & R. Sanderman. eHealth Research, Theory and Development: A Multi-Disciplinary Approach. Routledge. 2018.
43. Hekler EB, Klasnja P, Riley WT, Buman MP, Huberty J, Rivera DE, et al. Agile science: creating useful products for behavior change in the real world. Translational behavioral medicine. 2016;6(2):317-28.
84. Sauro J. A Practical Guide to the System Usability Scale: Background, Benchmarks & Best Practices. Scotts Valley, California, US: Createspace Independent Pub. 2011.
