# Supplementary material for: Combining Persuasive System Design Principles and Behavior Change Techniques in Digital Interventions Supporting Long-term Weight Loss Maintenance: Design and Development of eCHANGE
Source: JMIR Hum Factors. 2022 May 27;9(2):e37372. doi: 10.2196/37372 (PMC9187967; doi:10.2196/37372)
Supplement: Multimedia Appendix 4 [file humanfactors_v9i2e37372_app4.pdf]

MULTIMEDIA APPENDIX 4

Overview of end user (n=17) demographics.

**Characteristics**		
	**Categories**	**n (%)**
**Gender**		
	Male	5 (29)
	Female	12 (71)
**Age (years)**		
	20-35	2 (12)
	36-50	5 (29)
	51-59	7 (41)
	60-75	3 (18)
**Education level**		
	Higher education (>high school)	9 (53)
	High school	8 (47)
**Onset of obesity**		
	Child <12 years	4 (24)
	Youth 12-17 years	2 (12)
	Adult ≥18 years	5 (29)
	Not reported	6 (35)
**Employment status**		
	Working/studying full time	13 (76)
	Working/studying part time	2 (12)
	On disability	1 (6)
	Nonworking	1 (6)
**Use of health apps**		
	Yes	10 (59)
	No	7 (41)
