# Supplementary material for: Combining Persuasive System Design Principles and Behavior Change Techniques in Digital Interventions Supporting Long-term Weight Loss Maintenance: Design and Development of eCHANGE
Source: JMIR Hum Factors. 2022 May 27;9(2):e37372. doi: 10.2196/37372 (PMC9187967; doi:10.2196/37372)
Supplement: Multimedia Appendix 5 [file humanfactors_v9i2e37372_app5.pdf]

MULTIMEDIA APPENDIX 5 Virtual Coach and Smart, Tailored Feedback System (Examples)

Target area	Type of feedback	Examples	When
	**Animated Nudging Elements (eg, prompts)**		
Week 3	Animation - activity	All physical activity has health effect. Increasing your everyday activity can yield great results.	Every week (random when using the app)
Week 4	Animation - diet	Did you know that most people who achieve lasting weight loss start the day with breakfast?	Every week (random when using the app)
Week 5	Animation - wellbeing	Good and enough sleep is important. Sleep is maybe one of the most underestimated health habits. (See screenshot example Figure 8).	Every week (random when using the app)
Week 6	Animation - strategy	What motivates you to maintain weight after weight loss? Explore what is important to you in Knowledge and Skills.	Every week (random when using the app)
Week 7	Animation - activity	Regular physical activity prevents ailments and is an important resource in everyday life.	Every week (random when using the app)
Week 8	Animation - diet	Did you drink water today? Remember that water is the best thirst quencher.	Every week (random when using the app)
Week 9	Animation - wellbeing	Relaxation exercises can help you in a stressful everyday life.	Every week (random when using the app)
Week 10	Animation - strategy	Think about what you have achieved in the past - you can do it again!	Every week (random when using the app)
Week 11	Animation - activity	Take the stairs, do housework or take a walk in the neighborhood - all movement is good for the body!	Every week (random when using the app)
Week 12	Animation - diet	Eating regular, healthy meals helps against the feeling of hunger, nourishes and is good for your health!	Every week (random when using the app)
	**Rewards**		
Habit formation / rehearsal	Virtual coach - medal and confetti	Week goal! (See screenshot example Figure 8).	Goal achievement habits (ie, weekly and monthly close goal achievement)
	**Tailored Feedback**		
Habit goals	Virtual coach - praising message	Well done! You are good at making healthy choices.	Based on habit tracking
Sleep	Virtual coach	Good sleep is good for body and soul.	Based on sleep registration
Sleep	Virtual coach	Do you lack energy? Read about some of the tips about sleep in Knowledge and Skills.	Based on sleep registration
Mood	Virtual coach	What does usually help when you are in a bad mood? Sometimes it can be helpful to share thoughts and feelings with others. By checking the Knowledge part, you can find information about thoughts and feelings.	Based on mood registration
	**Feedback Goal Setting**		
Goal setting	Virtual coach - interactive response	Do you believe that you will achieve this? It is important that you are realistic and prioritize yourself.	When creating a Week plan (ie, healthy habit targets)
Goal setting	Virtual coach – interactive response	You have made a realistic week plan. Good luck!	Praising message based on individual scoring/reflection realistic goal setting (ie, Motivational Interviewing technique)

Target area	Type of feedback	Examples	When
	**Suggestion**		
Stress	Virtual coach	A simple advice for stress management is to breathe deeply and calmly with your stomach. Read more about stress management in Knowledge and Skills.	Based on stress level registration
	**Tailored Feedback**		
Weight: Green zone	Virtual coach - confetti	Great job!	Based on weight registration
Weight: Yellow zone	Virtual coach	What can you do to get back in the green zone again? Maybe looking at the Week Plan can be helpful.	Based on weight registration
Weight: Red zone	Virtual coach	What can help you when weight increases? Read about strategies and what you can do in Knowledge and Skills.	Based on weight registration
	**Praise Self-weighing**		
Weight registration	Animated effect - heart scale	See screenshot example in Figure 6.	Every weight registration
	**Reminder**		
Weight registration	Animated effect - champion	Weighing yourself regularly is a key to success! (See screenshot example Figure 8).	When no weight registration for 2 days
Weight registration	Push message	Remember to weigh yourself today!	When no morning weight registration for more than 2 days
App usage	Push message		
	**Praise**		
Healthy habits	Animated coach - praising message	Well done! You are great at making healthy choices!	Based on habit tracking / Close to target behavior
Healthy habits	Animated coach - praising message	This is great, you are well on your way to a healthier lifestyle.	Based on habit tracking / Close to target behavior
Healthy habits	Animated coach - praising message	Great! You are doing very well.	Based on habit tracking / Close to target behavior
Healthy habits	Animated coach - praising message	Hi! How are you doing with your habits? Maybe you can do some changes in the planning?	When no habit tracking
