# Supplementary material for: Combining Persuasive System Design Principles and Behavior Change Techniques in Digital Interventions Supporting Long-term Weight Loss Maintenance: Design and Development of eCHANGE
Source: JMIR Hum Factors. 2022 May 27;9(2):e37372. doi: 10.2196/37372 (PMC9187967; doi:10.2196/37372)
Supplement: Multimedia Appendix 6 [file humanfactors_v9i2e37372_app6.pdf]

MULTIMEDIA APPENDIX 6

Overview of *design features* reflecting the intervention content, PSD principles and BCTs combined and implemented in the eCHANGE intervention.

Main Intervention Components	Design Features	Design Feature Description	**Persuasive System Design (PSD) principles** from the PSD model(55)	**Behavior Change Technique (BCT) clusters and techniques** from Michie´s behavior change taxonomy(52)	**Key end user values(32)** V1: Personalized care, V2: Feel supported, V3: Positive self-image, V4: Health, V5: Happiness, V6: Motivation, V7: Autonomy, V8: Self-management.							
					**V1**	**V2**	**V3**	**V4**	**V5**	**V6**	**V7**	**V8**
Week Plan	(A) Animated onboarding	*Animated onboarding* provides an introduction to the app and guidance to create a personal *Week Plan* and *My Overview*. Individual preferences and self-determined goals can be selected during the onboarding to collect baseline data and shape the future interaction with the intervention (eg, how the content is delivered and visualized).	Tunneling<br>Personalization	**Goals and planning**	X						X	X
	(B) Behavioral planning and goal setting	The Week Plan supports creation of an *action plan* of healthy habits and personalized goals within the categories: 1) physical activity habits, 2) eating habits, 3) wellbeing (ie, sleep, stress management, mindfulness), 4) strategies. The *strategy* category includes maintenance strategies such as *problem solving/coping planning* to pre-plan for potential barriers and prevent relapse (eg, if-then plan, back on track strategy).	Reduction<br>Tunneling<br>Tailoring<br>Personalization<br>Suggestions	**Goals and planning**<br>1.1 Goal setting (behavior)<br>1.2 Problem solving/coping planning<br>(eg, for tempting situations, relapse prevention)<br>1.4 Action planning<br>1.5 Review behavioral goal(s)<br>1.6 Discrepancy between behavior and goal<br>**Shaping knowledge**<br>4.1 Instruction on how to perform a behavior<br>4.2 Information about antecedents	X	X				X	X	X
	(C) Motivational exercise and realistic goal setting	*Motivational exercise* supports realistic goal setting and creation of an achievable *Week Plan*. The interactive exercise (eg, self-evaluation bar 1-10) facilitates reflection and commitment to the plan.	Tailoring	**Goals and planning**<br>1.5 Review behavioral goal(s)<br>1.9 Commitment		X	X			X	X	X
	(D) Habit rehearsal and tracking	The *Week Plan* includes a self-monitoring tool for daily habit support to enable rehearsal and tracking of self-selected habits/healthy behaviors in a weekly or monthly overview (ie, calendar function). "My favorite tips" can be selected to support self-regulation and performance of the desired behavior.	Tailoring<br>Personalization<br>Self-monitoring<br>Rehearsal	**Feedback and monitoring**<br>2.2 Feedback on behavior<br>2.3 Self-monitoring of behavior<br>2.4 Self-monitoring of outcome of behavior<br>**Shaping knowledge**<br>4.1 Instruction on how to perform the behavior<br>4.2 Information about antecedents<br>4.4 Behavioral experiments<br>**Associations**<br>7.1 Prompts/cues<br>**Repetition and substitution**<br>8.1 Behavioral practice/rehearsal<br>8.2 Behavior substitution<br>8.3 Habit formation<br>8.7 Graded tasks	X	X	X				X	X

				**Reward and threat**<br>10.7 Self-incentive<br>10.9 Self-reward<br>**Antecedents**<br>12.1 Restructuring the physical environment<br>12.3 Avoidance/reducing exposure to cues for the behavior<br>**Identity**<br>13.4 Valued self-identity<br>**Self-belief**<br>15.3 Focus on past success<br>15.4 Self-talk								
**My Overview**	(E) Personalized self-monitoring	*Personalized self-monitoring* allows for registration of body weight, physical activity (ie, steps), perceived mood, stress, and sleep over time. Data can be entered manually and/or automatically (ie, activity tracker and weight) by personal choice and viewed in a weekly or monthly overview.	Personalization<br>Self-monitoring	**Goals and planning**<br>1.1 Goal setting (behavior)<br>1.3 Goal setting (outcome)<br>1.5 Review behavioral goals<br>1.6 Discrepancy between current behavior and goal<br>1.7 Review outcome goal(s)<br>**Feedback and monitoring**<br>2.2 Feedback on behavior<br>2.3 Self-monitoring of behavior<br>2.4 Self-monitoring of outcome(s) of behavior (eg, performance)<br>2.6 Biofeedback<br>2.7 Feedback on outcome(s) of behavior<br>**Natural consequences**<br>5.4 Monitoring of emotional consequences	X	X	X	X	X	X	X	X
	(F) Goal setting target outcome	*Goal setting* allows the user to set outcome goal (ie, weight target/weight maintenance goal). Actual weight or discrepancy from target weight (eg, +/- 3 kg) is visualized in *My Overview*.	Personalization	**Goals and planning**<br>1.1 Goal setting (behavior)<br>1.2 Problem solving<br>1.3 Goal setting (outcome)<br>1.4 Action planning<br>1.5 Review behavioral goal(s)<br>1.6 Discrepancy between behavior and goal<br>1.7 Review outcome goal(s)	X					X	X	X
	(G) Automatic integration of data	*Automatic exchange* and integration of data is available for activity tracking (ie, steps) and body weight (ie, through Apple Health and Google Health).	Personalization<br>Self-monitoring	**Feedback and monitoring**<br>2.6 Biofeedback	X			X	X	X		X

	(H) Visualization of target behavior in relation to target outcome	Target behavior(s) (ie, chosen habits) are visualized in relation to outcome (ie, weight) over time in a weekly or monthly overview, through graphs and icons in relation to weight zones (ie, green, yellow, red). Provides means for understanding the link between cause and effect of behavior and outcome to support self-regulation (eg, metacognition, behavioral awareness, self-reflection, comprehension). A progress bar (ie, a goal gradient effect) related to each habit, visualize adherence to the plan and behavioral performance in relation to targets and desired performance.	Personalization<br>Self-monitoring<br>Simulation	**Goals and planning**<br>1.5 Review behavioral goals<br>1.6 Discrepancy between behavior and goal<br>1.7 Review outcome goal(s)<br>**Feedback and monitoring**<br>2.2 Feedback on behavior<br>2.7 Feedback on outcome(s) of behavior<br>**Natural consequences**<br>5.4 Monitoring of emotional consequences<br>**Regulation**<br>11.2 Reduce negative emotions<br>**Identity**<br>13.3 Cognitive dissonance<br>**Self-belief**<br>15.3 Focus on past success	X	X	X	X	X	X	X	X
**Knowledge and Skills**	(I) Educational material and information	*Educational material and information* through 15 topics related to sustainable weight loss maintenance and behavior change. Provided through text or audio, and videos. The user can customize the content by marking and viewing "My favorites" only.	Tailoring<br>Personalization<br>Trustworthiness<br>Expertise<br>Surface credibility<br>Real-world feel<br>Verifiability<br>Social comparison	**Goals and planning**<br>1.2 Problem solving, coping planning (eg, barrier identification)<br>**Shaping knowledge**<br>4.1 Instruction on how (and when) to perform the behavior<br>4.2 Information about antecedents<br>4.3 Re-attribution<br>**Natural consequences**<br>5.1 Information about health consequences<br>5.6 Information about emotional consequences<br>**Comparison of behavior**<br>6.2 Social comparison<br>**Associations**<br>7.1 Prompt/cues<br>**Regulation**<br>11.2 Reduce negative emotions<br>11.3 Conserving mental resources<br>**Antecedents**<br>12.1 Restructuring the physical environment<br>12.2 Restructuring the social environment (eg, plan social support/social change)<br>12.3 Avoidance/reducing exposure to cues for the behavior<br>**Identity**<br>13.1 Identification of self as role model<br>13.3 Incompatible beliefs (including cognitive dissonance)<br>13.4 Valued self-identity	X	X	X	X	X	X	X	X

				**Self-belief**<br>15.3 Focus on past success<br>15.4 Self-talk								
	(J) Cognitive and motivational exercises	*Skills* training through 25 cognitive and motivational exercises to support self-regulation of behaviors, thoughts and emotions (eg, through self-reflection, metacognition), rehearsal of skills and strategies for relapse prevention, and continued motivation for sustainable behavior change (eg, identifying drivers, internal motivation, positive self-talk, focus on past success, coping planning, problem solving, stress management, mindfulness exercises).	Tailoring<br>Personalization<br>Rehearsal<br>Trustworthiness<br>Verifiability	**Goals and planning**<br>1.2 Problem solving, coping planning (eg, barrier identification)<br>**Shaping Knowledge**<br>4.1 Instruction on how to perform a behavior<br>**Associations**<br>7.1 Prompt/cues<br>**Repetition and substitution**<br>8.1 Behavioral practice/rehearsal<br>**Comparison of outcomes**<br>9.2 Pros and cons<br>**Regulation**<br>11.2 Reduce negative emotions<br>11.3 Conserving mental resources<br>**Antecedents**<br>12.1 Restructuring the physical environment<br>12.2 Restructuring the social environment (eg, plan social support/social change)<br>12.3 Avoidance/reducing exposure to cues for the behavior<br>**Identity**<br>13.1 Identification of self as role model<br>13.3 Incompatible beliefs (including cognitive dissonance)<br>13.4 Valued self-identity<br>**Self-belief**<br>15.3 Focus on past success<br>15.4 Self-talk<br>**Covert learning**<br>16.2 Imaginary reward	X	X	X	X	X	X	X	X
	(K) My favorites	*My favorites* is a general personalization feature that allows the user to mark and view only «My favorite» tips, skills training, knowledge, and/or strategies. *My favorites* can be selected and viewed separately for easy access, personalized content, and decision support.	Personalization<br>Suggestions	-	X					X	X	X
**Virtual Coach and Smart, Tailored Feedback**	(L) Virtual coach	A *virtual coach* (ie, animated coach/buddy) provides automated, tailored support (ie, smart feedback*) in a joyful way (eg, actionable feedback, motivating messages, prompt weight maintenance strategies, information about health effects, self-reward, or self-praise when reaching goals or performing target behavior). Adopts a social supportive role (eg, through motivating interviewing	Tailoring<br>Personalization<br>Social role<br>Social support**	**Goals and planning**<br>1.5 Review behavioral goals<br>**Feedback and monitoring**<br>2.2 Feedback on behavior<br>2.7 Feedback on outcome(s) of behavior)<br>**Social support**<br>3.1 Social support (unspecified)	X	X	X	X	X	X	X	X

		techniques). Provides real-time feedback on progress and performance of health maintenance behaviors based on; 1) outcome data: weight zones (ie, green, yellow, red zone in relation to target weight), 2) physical activity data from Apple Health and Google Health, 3) habit tracking/self-monitoring of behavior (eg, prompts close to target behavior/performance of habits), and 4) user data from the last 30 days (eg, feedback loops; prompts strategies for weight loss maintenance, motivational triggers for behavior change). In the settings of the app, type of feedback messages can be selected based on personal preferences.		3.3 Social support (emotional)<br>**Natural consequences**<br>5.1 Information about health consequences<br>**Associations**<br>7.1 Prompts/cues<br>**Repetition and substitution**<br>8.1 Behavioral practice/rehearsal								
	(M) Animated nudging elements	Animated elements are provided to prompt, encourage, and positively reinforce healthy behaviors and decisions to reach target goals/the desired behavior, through enjoyable and surprising animated elements. For example, a «heart scale» pop-up to encourage regular weight registration, animated effects (ie, firework/sparks) when habit tracking to stimulate adherence, animated follow-up prompts to elicit healthy behaviors and trigger performance of behavior).	Tailoring<br>Personalization<br>Reminders<br>Social role	**Feedback and monitoring**<br>2.2 Feedback on behavior<br>2.7 Feedback on outcome(s) of behavior)<br>**Social support**<br>3.1 Social support unspecified<br>3.3 Social support emotional<br>**Association**<br>7.1 Prompts/cues<br>**Repetition and substitution**<br>8.1 Habit formation<br>**Scheduled consequences**<br>14.4 Reward approximation		X			X	X		
	(N) Praise: Positive feedback	Praise is provided through positive, tailored feedback messages (eg, motivating messages by the virtual coach or animated elements/feedback messages) close to target behavior (ie, real-time), when reaching individual goals, and unexpectedly to stimulate motivation to sustain healthy lifestyle.	Tailoring<br>Personalization<br>Praise<br>Social support	**Feedback and monitoring**<br>2.7 Feedback on behavior<br>**Social support**<br>3.1 Social support (unspecified)<br>3.2 Social support (emotional)<br>**Associations**<br>7.1 Prompts/cues	X	X	X	X	X	X	X	X
	(O) Rewards	*Rewards* are provided by the Virtual coach when reaching (small) self-selected targets related to healthy habits to highlight goal achievement, facilitate engagement, and positively reinforce progress (eg, weekly and monthly reward). Achieved rewards can be viewed in a separate overview as part of the *Week Plan* (eg, self-rewards/self-praise strategies encouraged when a reward is achieved). Animated rewards (eg, sparkles) are provided when tracking performance of the behavior.	Personalization<br>Rewards<br>Liking	**Reward and threat**<br>10.2 Material reward (behavior)<br>10.9 Self-reward<br>**Scheduled consequences**<br>14.4 Reward approximation	X		X		X	X		
	(P) Reminders	*Reminders* are provided through "pop-up messages" (eg, on mobile device or compatible smartwatch) to facilitate engagement and adherence to the intervention and healthy lifestyle (eg, behavioral practice, weight registration, app use). Reminder settings can be personalized (eg, frequency and type of reminders).	Tailoring<br>Personalization<br>Reminders<br>Social role<br>Social support	**Feedback and monitoring**<br>2.6 Biofeedback<br>**Social support**<br>3.1 Social support unspecified<br>**Associations**<br>7.1 Prompt/cues	X					X		X

	(Q) Suggestions	*Suggestions* are provided by the virtual coach or through animated prompts/cues to support establishing and maintaining healthy lifestyle through various feedback messages (eg, suggestions of healthy habits, practical strategies in everyday life to keep weight off).	Tailoring<br>Suggestions<br>Social role<br>Social support	**Social support**<br>3.1 Social support (unspecified)<br>**Associations**<br>7.1 Prompts/ques<br>**Repetition and substitution**<br>8.1 Behavioral practice/rehearsal<br>8.2 Behavior substitution<br>8.3 Habit formation<br>8.4 Habit reversal<br>8.7 Graded tasks		X		X		X	X	X

**Data collected through self-monitoring or automatic trackers enables smart feedback and tailoring of the intervention content, related to behavioral performance and target outcome (eg, based on weight zones, traffic light system).*
***A general Social support (unspecified) principles was applied(35).*

References:

32. Asbjørnsen RA, Wentzel J, Smedsrød ML, Hjelmesæth J, Clark MM, Solberg Nes L, et al. Identifying Persuasive Design Principles and Behavior Change Techniques Supporting End User Values and Needs in eHealth Interventions for Long-Term Weight Loss Maintenance: Qualitative Study. Journal of medical Internet research. 2020;22(11):e22598.
35. Asbjørnsen RA, Smedsrod ML, Solberg Nes L, Wentzel J, Varsi C, Hjelmesaeth J, et al. Persuasive System Design Principles and Behavior Change Techniques to Stimulate Motivation and Adherence in Electronic Health Interventions to Support Weight Loss Maintenance: Scoping Review. Journal of medical Internet research. 2019;21(6):e14265.
52. Michie S, Richardson M, Johnston M, Abraham C, Francis J, Hardeman W, et al. The behavior change technique taxonomy (v1) of 93 hierarchically clustered techniques: building an international consensus for the reporting of behavior change interventions. Ann Behav Med. 2013;46(1):81-95.
55. Harri Oinas-Kukkonen MH. Persuasive Systems Design: Key Issues, Process
Model, and System Features. Communications of the Association for Information Systems. 2009.
