# Supplementary material for: Combining Persuasive System Design Principles and Behavior Change Techniques in Digital Interventions Supporting Long-term Weight Loss Maintenance: Design and Development of eCHANGE
Source: JMIR Hum Factors. 2022 May 27;9(2):e37372. doi: 10.2196/37372 (PMC9187967; doi:10.2196/37372)
Supplement: Multimedia Appendix 7 [file humanfactors_v9i2e37372_app7.pdf]

MULTIMEDIA APPENDIX 7
System Usability Check

System Usability Scale Results

The System Usability Scale (SUS) was applied(84), in combination with other formative evaluation methods (eg, think aloud), as a subjective measure of satisfaction with the system during the high fidelity prototyping and agile development process.

End users (n=8) reported an overall mean SUS score of 81.25, indicating a grade A, equivalent to *excellent* system usability, with a median SUS score of 87.50, indicating A+ or equivalent *best imaginable* system usability(84) (see Table A and Figure A below).

As indicated in Table A, overall mean score for health care personnel (n= 3) and healthy volunteers (n=5) was 74.17 and 73 respectively, equaling *good* system usability. The perceived usability score of the participants was relatively stable during the development process.

Table A. Results System Usability Scale

	**End users (n=8)**	**Health care personnel (n=3)**	**Healthy volunteers (n=5)**
Mean	81.25	74.17	73.00
Median	87.50	77.50	72.50
St. deviation	19.32	5.77	13.28

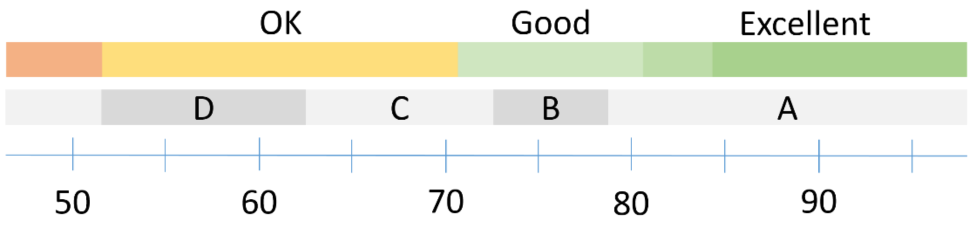

Figure A. System usability scores and grading A-D. Values 84.1-100 are *best imaginable* (ie, grade A+) and values 80.8-84.0 are *excellent* (ie, grade A). Values lower than 51.6 are *poor*.

Reference:

84. Sauro J. A Practical Guide to the System Usability Scale: Background, Benchmarks & Best Practices. Scotts Valley, California, US: Createspace Independent Pub. 2011.
